# Smart Hydrogel Architectures for Sensors: Narrative Review

**DOI:** 10.3390/s26103213

**Published:** 2026-05-19

**Authors:** Jūratė Jolanta Petronienė, Tadas Rasimavičius, Darius Viržonis, Andrius Dzedzickis, Vytautas Bučinskas

**Affiliations:** Department of Mechatronics, Robotics and Digital Manufacturing, Vilnius Gediminas Technical University, Plytinės g. 25, LT-10105 Vilnius, Lithuania; tadas.rasimavicius@vilniustech.lt (T.R.); andrius.dzedzickis@vilniustech.lt (A.D.); vytautas.bucinskas@vilniustech.lt (V.B.)

**Keywords:** carbon nanomaterials, force sensor, conductive hydrogel, hydrogel architecture, polymer, multifunctional sensor, 3D printed hydrogel

## Abstract

In sensing technologies, a hydrogel sensor with a specific response to stimuli allows for real-time monitoring of mechanical, thermal, and biochemical signals in wearable and implantable devices. This review discusses the latest advances in hydrogel-based sensors published between 2023 and spring 2026 and the design strategies prevalent in these articles, including the use of polymers, nanomaterial reinforcement, incorporation of ionic solvents, and physical or chemical crosslinking. The influence of supramolecular hydrogels on the quality of sensor parameters, including the impact on mechanical resistance, ionic conductivity, adaptation, and self-healing, is examined. In biomedical engineering, hydrogels, thanks to their biomimetic and programmable properties, enable control of wound repair and soft tissue interfaces. The review concludes by outlining the challenges, opportunities, and advances in the chemistry and mechanics of hydrogels, which may ultimately facilitate the development of multifunctional monitoring systems in healthcare. The abundance of information requires systematic, frequent reviews to accelerate the application of innovative solutions in practice. Carbon nanostructures are a key component that ensures the sensor’s electrical conductivity. 3D printing technology has enabled the creation of individually customizable health monitoring devices. The work also highlights the use of nanodots in sensor production.

## 1. Introduction

Modern smart human health monitoring [1,2] has become a cornerstone for the next generation of healthcare. Moreover, health monitoring devices have become increasingly multifunctional, enabling a broader range of applications. More desirable devices not only perform the functions of a mechanical sensor but also act as biosensors. The need for continuous, non-invasive, and personalized physiological assessment drives this duality. A separate class of devices is labeled implantable biodegradable sensors, which are very important for postoperative monitoring to avoid additional manipulations during the removal of a redundant implanted sensor. Implantable sensors and biosensors, as essential medical devices, are designed to monitor physiological signals in vivo. Still, their invasive nature often poses risks of tissue damage and biocompatibility issues for patients.

With the advent of 3D printing technologies, there is increasing interest in developing a one-piece, flexible hydrogel-based sensor [3] and biosensor [4] tailored to the individual needs of specific patients. Sensor developers report the successful application of a new material or even an entirely new class of materials, bringing enormous benefits to the entire sensor development process. With the first successful applications of conducting polymers or hybrid composites in the early nineties of the last century, key sensor performance parameters such as sensitivity, mechanical durability, and strain-sensing range were significantly improved [3]. This has led to more accurate working sensors and, at the same time, wider applications and greater reliability. The importance of interdisciplinary collaboration to fully realize the potential of flexible strain sensor technologies in adaptive, high-performance systems is evident [4,5].

The most relevant topics regarding possible directions for sensor development include the following: improving electrical conductivity, mechanical strength, and long-term stability under difficult operating conditions, and transforming existing compositions by introducing an additional chemical element into various polymers or carbon nanostructures [6]. Next-generation flexible designs are enabled by advanced materials and additive manufacturing using 3D printing [7]. In general, conductive hydrogel (CH)-based electrochemical/chemical sensors are attractive due to their soft interface that is compatible with living tissues. However, the soft interface can overcome the mechanical mismatch and signal instability associated with traditional rigid electrodes.

When discussing the importance of hydrogels in the production of healthcare devices, we must evaluate the types of hydrogels and their specific uses. Conducting hydrogels are promising in the field of health monitoring, in practically all possible areas of medical activity, due to their unique properties, such as medically appropriate electrical and optical properties, physical and chemical stability, high conductivity, and efficient redox properties, characterized by high temperature stability and biocompatibility [8].

This review provides a comprehensive overview of the role of hydrogels in sensor development and their applications in implantable or health-monitoring devices, emphasizing the contributions of microstructures, micropatterns, micropores, and hierarchical structures in optimizing performance. To demonstrate the versatility of these sensors, specific application areas are discussed, including human physiological signal monitoring, motion detection, soft robotics, and emerging applications.

## 2. Methods

The development of technologies for creating health-monitoring sensors based on natural hydrogels creates unique opportunities to address challenges such as product compatibility with the human body, reduced technology costs, and reduction of waste unsuitable for recycling. The collected information is presented as a narrative review with the authors’ viewpoint. Reliable data were obtained after a three-stage procedure: sorting by keywords and article title; screening; and detailed reading in two stages; abstract reading and whole-article reading. This selection identified the most frequently recurring topics and highlighted new but less popular ones. The focus was on a few main topics: hydrogel-based sensors for health monitoring and flexible sensors for professional medical care, as well as the influence of renewable resources on modern technologies. Selected information highlights the diversity of solutions, promising outcomes, and cutting-edge research methods. The work does not discuss hydrogel-based sensors whose structure does not use nanomaterials. The search keywords for this study data were biodegradable, biomedical, biopolymer, carbon nanomaterial, carbon dots, sensor, hydrogel, and 3D printing.

After presenting information gathered from scientific articles, we will discuss the following topics:

- Hydrogel combinations with carbon nanomaterials for force sensors in the field of medicine—recent reports.
- Nanomaterial-based sensing: application of carbon nanomaterials, MXene, metal-organic frameworks, plasmonic nanoparticles, quantum dots, and magnetic nanoparticles to increase sensitivity and enable new transmission mechanisms due to low detection limits down to single molecules, selectivity, and integration into implantable formats.

The review is limited to the period from 2023 to April 2026—information was collected from MDPI, Google Scholar, ScienceDirect, and Wiley Online Library sources.

## 3. Hydrogel Application for Sensors

Development and designing of hydrogel-based sensors often involves a trade-off between mechanical robustness and multifunctional stimulus-responsiveness [9], and use of traditional threshold analysis methods [10,11]. They are difficult to interpret accurately without modern machine learning and artificial intelligence [12], one of the methods for controlling hydrogel physical parameters. Therefore, one of the challenges in developing hydrogel-based sensors is accurately interpreting the collected data using the latest technological capabilities. The CH offers the possibility to combine the biomimetic properties of hydrogels with the functionality of conductive components, becoming a very attractive material combination for many scientific fields and giving strong impetus to the development of technologies, especially in biomedical engineering [13].

Hydrogel-based systems face significant obstacles: insufficient electrical energy [14,15] to meet physiological needs [16], influences of inherent fragility, swelling-induced degradation of mechanical and functional properties in humid environments, and incompatibility with established electronic manufacturing, hindering precise integration, as Ch. Zhi [17] states.

There are over 6000 publications published in 2025 dedicated to improving hydrogels by monitoring ionic conductivity [18], but only a little over a thousand are dedicated to the targeted use of these hydrogels in sensor development, of which only a few hundred publications match our chosen keyword combination. Searching for published knowledge using the keywords “hydrogel; sensor; 3D printing” yielded 2.6 thousand works, of which only 1736 are research papers. Searching for the keywords “hydrogel, ionic conductivity, force sensor” returns 1196 publications from 2025, 838 publications from 2024, and 510 from 2023. Based on data from April 2026, 725 publications have already been published in 2026, of which 289 are review publications, and 357 are research publications.

Hydrogels suitable for health status monitoring sensor manufacturing can be divided into two main groups: natural origin hydrogels and synthetic hydrogels. Natural hydrogels are normally more biocompatible and biodegradable. Due to their mechanical properties, such as a specific range of stiffness and tensile strength, the hydrogels have specific fields of application. Synthetic hydrogels exhibit better mechanical stability; however, they tend to be less biocompatible [19]. The physical crosslinking types, hydrophobic interactions, host–guest interactions, and detailed analysis of chemical crosslinking are presented by X. Cao [20]. Intermolecular interactions and hydrogen bonds play a key role in strengthening the interface bonds of nanoparticles (NP). In the nanocomposite hydrogel system, the large specific surface area of NPs increases the number of potential hydrogen-bonding sites; therefore, the surface-modified NPs can be considered multi-level supramolecular cross-linking centers, allowing simultaneous interaction with multiple polymer chains through hydrogen bonds [20]. Hydrogels formed via hydrophobic interactions have also been widely studied, typically containing numerous hydrophilic macromolecular chains and hydrophobic chain segments that serve as cross-linking points within the polymer network [21]. Physically crosslinked hydrogels can be called “reversible hydrogels” because they dissolve in response to environmental variables such as pH, ionic strength, and temperature, thereby facilitating modulation of hydrogel structure and dissolution. Chemically crosslinked hydrogels offer distinct functional profiles and broader application potential than physically crosslinked hydrogels, as their main structural blocks are linked by covalent bonds, which confer a high density of crosslinks and, therefore, high resistance to degradation. Interpenetrating polymer networks (IPN) are three-dimensional architectures composed of two or more chemically distinct polymer networks intertwined at the molecular level, without covalent bonds between them [22]. These structures differ from blends or graft copolymers in that they exhibit improved stability and performance. Systems consisting of a physically crosslinked network and a separate chemically crosslinked polymer network are classified as hybrid physicochemical dual networks rather than true IPNs [22]. Multifunctional hydrogels [23] are widely used in smart perception [14], real-time health monitoring [14], rehabilitation training [24], entertainment virtual reality [25,26] and augmented reality technologies [27], providing people with a more convenient and comfortable way to monitor their daily health status [16]. To overcome the limitations of hydrogels [25,28,29]. Q. Wang [30] presents a multifunctional hydrogel composite fabricated via a synergistic strategy that combines cyclic freeze–thaw and salting processes. The most important innovation of this work is the engineering of a gradient concentration distribution of AgNWs within a PVA matrix, which enables adjustment of strain sensitivity. This gradient nanoparticle arrangement gives the hydrogel an exceptional negative resistance-to-strain ratio and widely tunable up to GF = 983. An important fact is that this material is recyclable—it retains more than 90% of its original properties after recycling and reprocessing.

The most common cause of loss of physical properties of a hydrogel is the removal of water molecules from the hydrogel structure. To prevent water loss in hydrogels, various strategies have been developed to improve their water retention through multiple mechanisms and across multiple application areas. The review of Y. Chang [31] systematically describes the strategies to improve the water retention of hydrogels and their corresponding state-of-the-art applications, formulates the states and importance of water in hydrogels, discusses the main strategies, categorizes them and analyzes them mechanistically at various levels: encapsulation, solvent optimization, ion incorporation, structural design and combined methods; highlights the application areas and development of hydrogels, mainly divided into three promising candidates, including biomedical fields; critically evaluates the current challenges and future research directions of hydrogels; and emphasizes the need for comprehensive solutions and strategic advancements to fully exploit their potential in various fields.

Y. Li reports the smart application of a PVA-based hydrogel embedded in artificial skin [32]. It provides a detailed analysis of how different crosslinking strategies affect the network structure and performance of PVA hydrogels. It also reveals the role of crosslinking mechanisms in regulating key properties such as conductivity, mechanical properties, self-healing ability, and swelling resistance [33,34,35,36,37,38,39]. Ch. Liu [40] presents an in situ ion pre-charging method for fabricating hydrogel networks and increasing the number of cross-linking sites to maintain uniform ion distribution. Notably, this strain sensor achieves 99.06% accuracy in motion digit recognition, as validated using machine learning.

T. Zheng [41] reviews systematically synthesis mechanics-oriented design strategies: dual-network architectures, ordered structural motifs, and regulation of reversible physical interactions. Although many literature reviews are currently available for discussion, each author evaluates publications from a particular aspect or perspective. Among the multitude of thematic publications, a particular approach by review authors can be useful for both developers and product users who follow innovations. The synthesis techniques, biomedical functionalities, and engineering challenges are discussed in the V. Ramkumar [42] review. Synthesis methods of biologically responsive hydrogels and their limitations, the role of nanomaterials/hydrogel-based biosensors in diagnostics and health monitoring, and opportunities and challenges for use in implantable, wearable and disposable biosensors are discussed by A. Barhoum [43]. Hydrogels as thermo-responsive material suitable for multifunctional applications are presented by J. Farahbakhsh [44]. A. Ramesh [45] discussed the stages of hydrogel production, their properties, and applicability for medical purposes, paying little attention to the issue of hydrogel sensors.

Shape memory hydrogels (SMHs) are a promising class of smart materials that have attracted considerable attention across various fields, including biomedical engineering, soft robotics, sensors, and mechanical systems, owing to their ability to recover a predefined shape in response to external stimuli such as temperature, pH, or visible light. They found applications in sensor and actuator manufacturing, biomedical domains, energy storage, and wearable electronics. Despite their potential, several challenges prevent them from moving from laboratory research to industrial use. The main drawback of SMH is its fatigue resistance [46]. According to publication statistics, 75 research articles on “Shape memory hydrogel” were published during the period under study, and 110 reviews were published. Thus, it fully aligns with the general publication trend, in which more attention is paid to finding solutions than to publishing successful research.

### 3.1. Supramolecular Chemistry and Hydrogel Sensors

When it comes to hydrogels, it is necessary to discuss supramolecular chemistry as a science combined with nanotechnology, which has fundamentally changed the design of hydrogel-based soft electronics by integrating dynamic molecular interactions with nanoscale conductive architectures. Hence, supramolecular hydrogels, stabilized by reversible non-covalent interactions, exhibit self-healing, adaptive, and biocompatible properties that are essential for interaction with biological systems [47]. M. Mascini [48] presents advances in conductive polymer architectures, including nano-capsulation for clinical applications. The number of research publications on the topic of “supramolecular hydrogel” sensor reaches 320 in 2025, 270 in 2024, and 223 in 2023. Y. Song [49] presented a structurally refined cellulose framework as a reinforcement template. A cellulose framework-reinforced ionic CH (PADW4-Li) was fabricated via photoinitiated polymerization of acrylamide monomers and demonstrated excellent adaptability, enabling its assembly into high-performance sensors that monitored physiological signals and exhibited high sensitivity to small deformations. A unique work by Y. Zeng [50] presented a supramolecular CH named silk fibroin/wool keratin/tannic acid/multi-walled carbon nanotubes with shape plasticity. In particular, wool keratin not only induces a secondary-structure transition in SF from random coils to α-helices and β-sheets but also synergistically disperses MWCNTs with TA through strong π–π interactions. As a result, SWTN exhibits excellent strength (578.3 kPa) and optimized conductivity (0.073 S/m). Furthermore, the conductive SWTN hydrogel exhibits competitive properties for large-strain (up to 350% elongation) and cyclic-compression (10 cycles) sensing, making it an effective strain sensor for sensitive, stable, and accurate monitoring of human activities such as finger flexion. The supramolecular gels reported by X. Chen [51] consist of 5,10,15,20-tetra-(4-carboxyphenyl)porphyrin and Li+ ions, forming a rigid, interconnected network via ref. [52]. The introduction of PVA improves the gel’s flexibility by dissipating energy. The interaction of the rigid and flexible networks increases the mechanical strength of the PVA/TCPP/LiCl supramolecular gel. The hydrogel exhibits excellent self-healing properties, strong adhesion to various hard and soft substrates, maintains softness at −40 °C, is thermally stable, protects against UV radiation, and is electrically conductive. These properties can be applied to flexible sensors for monitoring human movements. T. Lei [53] presented a hydrogel strain sensor with strain sensitivity (GF = 2.01) and a wide sensing range from 1% to 850%, where excellent properties were elaborated due to the addition of functional ionic liquids, 1-vinyl-3-butylimidazolium bromide and ionic salts (AlCl_3_); the hydrogel exhibited excellent ionic conductivity (21.65 mS/cm), anti-freeze properties (−32 °C) and water retention. With the addition of tannic acid, the hydrogel featured a catechol moiety, thereby enabling excellent adhesion. The work of Z. Tang [53] presented a supramolecular conductive PVA/TA hydrogel, which exhibits excellent electrical properties with a conductivity of S = 5.5 × 10^−4^ S/cm and GF = 1.3 under dry conditions and S = 5.0 × 10^−4^ S/cm and GF = 1.2 under wet conditions, respectively, as well as a tensile strength of 700 kPa and an elongation of 4700%. Due to their excellent swelling resistance and stability, high electrical conductivity, and extraordinary stretchability, the hydrogels are promising for use in amphibious motion sensors. In the work of F. Xia [54], the polymer CS-P(AM-G) was prepared by reversible addition fragmentation chain transfer polymerization—chitosan grafted with poly(acrylamide-1-vinylimidazolium bromide) (P(AM-G)). The three-dimensional porous structure of the CS-P(AM-G)@CB supramolecular hydrogel confers antibacterial properties and good electrical conductivity, and it can monitor real-time changes in body movements (fingers, wrists, elbows, and knees). In the work of S. Li [55], inspired by the lipid structure of cell membranes, an aramid fiber (ANF) reinforced poly(vinyl alcohol) (PVA)-based organic ion-conducting hydrogel membrane (PVA-ANF hydrogel) was fabricated by adopting a dynamic cross-linking and hydrogen bond network cooperation strategy, with a conductivity of 1.019 S/m, which enabled accurate detection of human body movements and small movements such as impulse with a strain sensitivity of 0.2%. D. Fang [56] reported a zwitterionic lignin that promotes supramolecular interactions among lignin, polymer chains (poly-(acrylic acid) and gelatin), and adhesive substrates; internal cross-linking and external adhesion are enhanced simultaneously. Notably, the hydrogel exhibits a remarkable true stress of 2.34 MPa at a strain of 1597.1%, and its peak adhesion strength is 40.87 kPa.

The Fe_3_O_4_ hybrid supramolecular hydrogels were fabricated via dynamic host–guest interactions between the host molecule cucurbit[6]uril-modified (CB[6]) and guest units. The “supramolecular crosslinker” formed by CB[6]-modified Fe_3_O_4_ (Fe_3_O_4_@CB[6]) nanoparticles, was used to generate a 3D network of supramolecular hydrogels in the Y. Yang [57] work.

The Fe^3+^-mediated physical crosslinking is employed to construct chemically crosslinked poly(acrylamide-*co*-acrylic acid) networks for a multifunctional sensor made from wood-based hydrogel and named as soft- and-wet material with a tensile strength of 42 mPa, elaborated by L. Wang [58].

### 3.2. Types of Hydrogels in Sensor Manufacturing

Self-healing hydrogels typically contain a large number of water molecules, which make them adhesive and biocompatible. However, such hydrogels lack conductive structures due to the relatively low number of conductive ions, and thus, self-healing hydrogels have the inherent problem of low ionic conductivity, which limits their applications in actuators and high-sensitivity sensors [59].

**Figure 1:**
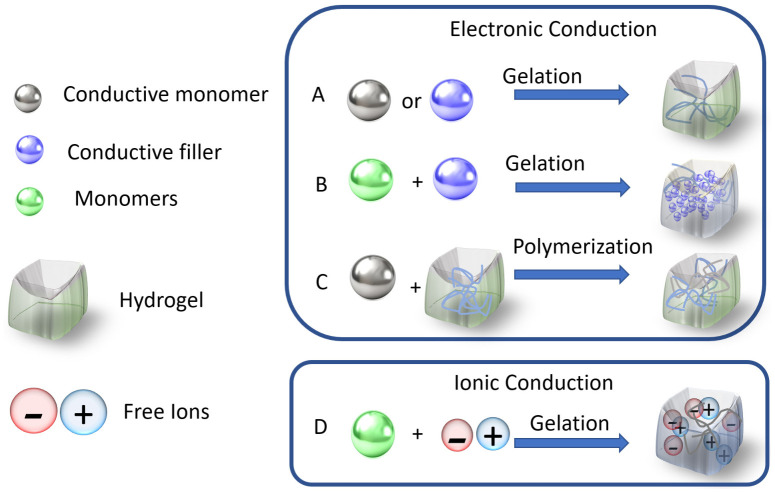
Schematic representation of charge transfer mechanisms in conductive hydrogels: (**A**) direct gelation by cross-linking agents, (**B**) conductive fillers within the hydrogel matrix, (**C**) in situ polymerization of the hydrogel network, and (**D**) achieving ion-mediated conductivity.

In electronically conductive systems, charge flows through networks of conjugated polymers or dispersed conductive fillers, allowing electrons to permeate the hydrogel matrix. Charge transport pathways depend on π–π interactions, tunneling effects, or direct contact between conducting domains. In conducting hydrogels, charge is transported by electronic and ionic conduction. The single network type hydrogels can incorporate conductive fillers [60]. Electronic conductivity provides higher conductivity and stability, while ionic conductivity systems provide better biocompatibility. Therefore, balancing these charge transport mechanisms (Figure 1) can create multifunctional hydrogels for flexible electronics, biosensors, and soft control applications [47,61]. The self-healing polymers are another stimulus-responsive hydrogel [62]. Modern products are not limited to the need for a multifunctional sensor. A recent M. Beg [63] article reports on research into a low-voltage, high-capacity sweat-activated tissue battery, a biomaterial-based piezoelectric hydrogel that can be used for energy storage, along with force and bending sensing, for wearable and biomedical devices. The B. Song [64] review outlines the design path of hydrogel electronics across various directions: molecular strategies, microstructural architectures, integration of macroscopic functions or mechano–electro–thermo–chemical responses, and system-level considerations. The S. Choudhury [65] review discusses aspects of hydrogel material application for implantable bioelectronics.

Plant polyphenol-based hydrogels exhibit good adhesion, antibacterial activity, self-healing, and biocompatibility, and their great potential in the biomedical field is discussed in the Y. Chen review [66].

To fully exploit the potential of ionic hydrogels, the challenge for researchers involves creating stable bonds between hydrogels and rigid external chains without the use of toxic additives [67]. In the ionic CH, the incorporation of free mobile cations, such as Fe^3+^, Ca^2+^, Li^+^, and anions like SO_4_^2−^ and Cl^−^, significantly increases the conductivity of the hydrogel or charge transfer [68]. L. Zeng [69] states that excessive Fe^3+^ ions disrupted the crosslinking of the hydrogel. Y. Li [70] presented PAAm/PVA composite with tannic acid-modified lignin and Fe^3+^ post-impregnation and elaborated a hierarchical triple-network structure for a strain sensor, where the covalently crosslinked PAAm backbone in the PVA network and metal-phenol coordination interactions represent hydrogel stability.

In the work of C. Lee [71], the effect of different ratios of Fe^3+^ ions to DA on the performance of each hydrogel was investigated. The results show that incorporating iron–dopamine complexes significantly enhances the hydrogel’s mechanical strength. X. Guan [72] synthesized a dual-conductive lithium-ion and calcium-ion hydrogel based on acrylamide/gelatin using a simplified low-temperature one-step polymerization method. The results show the broad application potential of the hydrogel in wearable sensors, ecological monitoring, and smart agriculture.

When designing structures in non-ionic, conductive hydrogels, the resulting conductivity often conflicts with the material’s mechanical properties, making it difficult to design hydrogels that balance conductivity and mechanical performance. J. Ren [73] solved the problem of hydrogel stability by integrating CNT layers into a sandwich structure on the surface of a cationic hydrogel (P(AM-co-DMDAAC), PAD) and densified it by controlled dehydration.

The sol-gel hydrogels are programmable; once created, they can change shape among a set of equilibrium shapes, including swelling/deswelling. The topic “sol-gel hydrogel” attracted attention in 2025, with the following article distribution: 430 review articles and 302 research articles according to ScienceDirect statistics, and 126 in MDPI search results, most of which are research papers. Z. Jiang [74] presented the preparation of ion-thermoelectric gels for a strain sensor, withstanding 120 cycles; GF varies from 0.92 to 2.15, and depends on strain ranges from 50% to 320%, respectively.

The application of alginate-based hydrogels for various purposes, including force sensors, is discussed in detail by X. Liang [75], summarizing advances in key structural properties, main crosslinking methods—ionic coordination with divalent cations (Ca^2+^, Ba^2+^, Sr^2+^), covalent chemical bonds, and hybrid multi-crosslink systems—and strategic modification strategies including chemical derivatization, polymer blending, and nanoparticle incorporation. The L. Guan [76] review systematically explores the complex relationships between the composition, structure, and fabrication strategies of CH, while highlighting their recent achievements in biomedical applications, including skin tissue regeneration, spinal cord injury repair, muscle tissue reconstruction, cardiac tissue engineering, and biosensor technologies—the design and synthesis of various CHs, the current limitations of CH’s practical applications, the prospects for future development directions, and the interplay between material properties and biological functionality are analyzed, and their application in next-generation biomedical innovations is explored. The Ch. Shen [77] review examines 3D bio-printed hydrogel-based implantable electronics for sensing and stimulation, regenerative therapy, and discusses immuno-compatibility, mechanical stability, and long-term functionality, as well as strategies to accelerate clinical application and increase the functional versatility of hydrogel technologies.

It is worth noting in the review of K. Zhang [78] that the principles of eutectic-gel design, material selection, sensing mechanisms, and flexible sensing applications are discussed, as well as that the design of eutectic-gels, DES selection, and gel network synthesis are examined, and a theoretical basis for the development of sensors based on eutectic-gels is also provided. The review of L. Sun [79] provides a comprehensive overview of the controlled synthesis of chiral plasmonic nanomaterials and their biomedical applications for chirality-dependent enantioselective recognition, biosensors, and therapeutic interventions, as well as recent developments in this area, especially those with intrinsic structural chirality. This publication discusses in detail the novel biomedical applications of chiral plasmonic nanomaterials, their main challenges, and future research directions. These studies should provide new insights into the interactions of artificial chiral materials with hydrogel-based biological systems.

### 3.3. Nanomaterials in Hydrogel-Based Sensor Mmanufacturing

When discussing materials that can substantially improve the performance of hydrogel sensors, we must mention MXenes; they are a family of 2D-structured inorganic nanomaterials composed of atomically thin layers of transition-metal carbides, nitrides, or carbonitrides. The enrichment of the hydrogel structure with MXene has been reported in more than 800 scientific papers in 2025, of which only 280 are research articles. Many MXenes are transition-metal carbides or carbonitrides, in which carbon is an essential part of the X layer of their structure. The incorporation of functional nanomaterials, such as MXene and metal–organic frameworks, into hydrogel-based sensors has expanded their capabilities by enhancing electrical conductivity, catalytic activity, porosity, and analyte selectivity. These hybrid hydrogel architectures exhibit improved charge-transfer performance across a variety of health-monitoring applications, from metabolite and biomarker detection to wearable and implantable sensing platforms [29,80,81]. Z. Li [82] presented loofah-inspired PPy@BC/MXene/PVA hydrogels that form anisotropic fiber-skeleton networks with high strength and toughness, where well-dispersed Ti_3_C_2_T_x_ MXene is incorporated into a hierarchically aligned porous architecture. Mxene-based pressure sensor manufactured using PVA/H_2_SO_4_ hydrogel as the electrolyte and dual-functional cross-linker Zn(BF_4_)_2_ was elaborated by H. Fu [83] and delivers outstanding areal capacitance.

To solve the dilemma of hydrogel brittleness, J. Shi [84] designed and prepared a macromolecular polyurethane crosslinking agent (PCA) in his work; then, PCA and two-dimensional (2D) MXene nanosheets were introduced into a covalently linked network to improve the comprehensive mechanical and electrochemical properties of the hydrogels. Two-dimensional MXene nanosheets provide the hydrogel with high electrical conductivity and strain sensitivity, making it a wearable device for continuous monitoring of human movements and facial micro-expressions. This study demonstrated an effective structure design strategy for creating mechanically robust, highly stretchable, and sensitive dual-mode MXene-based wearable sensors. MXene-polymer nanocomposite hydrogels have become the basis for a new generation of wearable sensors that offer an exceptional combination of mechanical strength, high electrical conductivity, and responsiveness to a variety of external stimuli [85]. A. Arinova [86] presented PAM (polyacrylamide)-Ag-g/WS_2_/Ti_3_C_2_T_x_ hydrogel for strain sensor manufacturing.

The Annu [87] review discussed chitosan-MXene hybrid materials, which have exceptional potential for fabricating healthcare sensors and are suitable for smart wearable bioelectronics, where simultaneous cytocompatibility, signal transduction, and mechanical adaptation are required. It is also emphasized that incorporating MXene significantly improves ionic/electronic conductivity, sensory sensitivity, physiological adhesion, hemostasis, and near-infrared sensitivity. At the same time, chitosan regulates biodegradation, reduces cytotoxicity, and improves physiological stability. The work highlights novel fabrication strategies, including in situ nanolayer dispersion, dynamic covalent crosslinking, ionically coordinated networks, and photothermal self-gelation, which provide precise control over network architecture and enable customized design.

**Figure 2:**
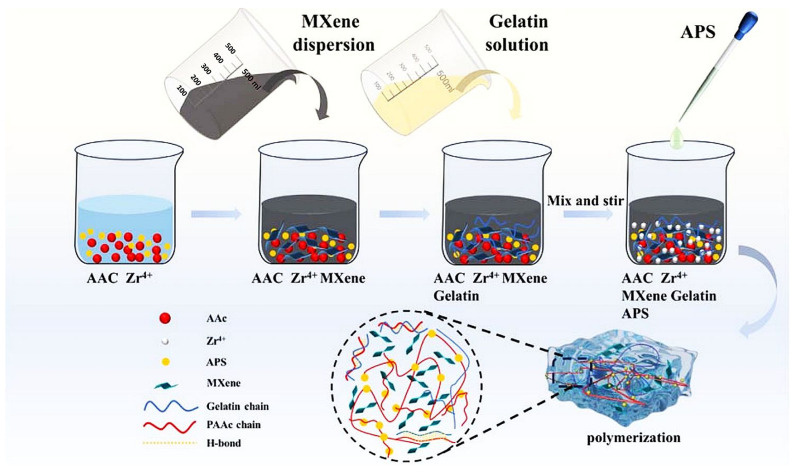
Schematic of the MPG hydrogels preparation process and the polymerization mechanism from ref. [88].

Q. Wei [88] reported that incorporating Zr^4+^ coordination crosslinking created a robust “covalent-physical-coordination” triple network (Figure 2). This structure not only provides the hydrogel with excellent mechanical resistance but also significantly limits water absorption, ensuring structural stability in a humid environment. A smart insole sensing system was developed. Leveraging the high signal-to-noise ratio and temporal stability of MPG sensors, the proposed CNN-LSTM hybrid deep learning model effectively extracts spatial and temporal features from complex gait signals. Y. Li [89] elaborated a conductive double-network hydrogel composed of PAM and CMC, synthesized by a one-step strategy in a binary solvent of glycerol and water as a rigid primary network and a secondary network synthesized through divalent chelation with Ca^2+^. Obviously, the incorporation of glycerol provides exceptional resistance to freezing and desiccation, maintains stability over a wide temperature range, and is suitable for 3D bioprinting. Notably, the electromagnetic shielding efficiency of the MXene (Ti_3_C_2_T_x_)-embedded hydrogel reaches 46.3 dB. S.-Ch. Tan [90] presented a PAA nanocomposite hydrogel strain sensor by incorporating Pt NPs modified Mxene and tannic acid into the hydrogel network, denoted as PAA-TA@Pt@MXene.

Tan. W. [91] presented conductive hetero-structural nanocomposite WS_2_/Ti_3_C_2_T_x_, available to improve mechanical properties of hydrogels.

The work of Y. Zhang [92] draws attention to a special combination of materials and states that PVA and ferric chloride contribute to the mechanical strength of the composite hydrogel. Still, ferric chloride significantly reduces hydrogel elongation, while PVA’s effect on elongation is relatively small. The energy dissipation capacity of physical hydrogels increased significantly with increasing ferric chloride content, because the increased Fe^3+^ content could form more physical cross-linking points and contribute to energy dissipation during the elongation process; the increase in PVA had a smaller effect on energy dissipation, and even with a higher Fe^3+^ content could form more physical cross-linking points and contribute to energy dissipation during the elongation process; the increase in PVA had a smaller effect on energy dissipation, and even with a higher Fe^3+^ content, the energy dissipation decreased with increasing PVA content, probably because PVA hinders the movement of polyacrylic acid chain segments, thus preventing the disruption and reorganization of the interaction structure between Fe^3+^ and carboxyl ions, as explained by the authors. L. Zeng [69] presented injectable CH synthesized from oxidized dextran, chitosan, and Fe^3+^ as a wound healing and strain sensor with GF from 2.13 to 2.76. The copolymerization of carboxyl-group-functionalized CNT-encapsulated liquid metal and polyacrylamide/sodium alginate hydrogel resulted in PSCLE hydrogel fibers for human motion observation [93].

As one of the essential strategies for changing the structure of a material, Y. Huang [94] describes crosslinking strategies and methods of fabrication of nanofibrous hydrogels, discusses the mechanical, electrical, self-healing, antibacterial, and biocompatible properties of nanofibrous hydrogels, presents several promising applications of nanofibrous hydrogels in the field of energy storage and wearable sensors—emphasizing durability and adaptability under harsh conditions—provides a comprehensive overview of the design principles and synthesis strategies of nanofibrous hydrogels, as well as a detailed analysis of their main characteristics.

As one of the possible strategies for improving hydrogel properties, M. Z. Anwar [95] discussed hybrid hydrogels that had reached clinical evaluation. The article also examines physicochemical crosslinking strategies, lipophilic and hydrophilic drug loading with nanoparticles, and basic design principles for the fabrication of multifunctional hybrid hydrogels, recent advances in incorporating various nanoparticles, such as polymeric nanoparticles (NPs), metallic NPs, carbon-based NPs, lipid-based NPs, dendrimers, and nano-emulsions, into hydrogel matrices, with emphasis on drug release mechanisms and biomedical applications. The hydrogel crosslinking approaches and controlled radical polymerization in hydrogel structure manufacturing are described in detail by D. M. Ampong [96]. A dual-mode electrical–optical nanocomposite hydrogel, presented by Ch. He [97] was fabricated by integrating carboxyl-modified conversion nanoparticles (UCNP-COOH) and quaternized chitosan (CQAS) into a covalent PAAm network. The hydrogel exhibits high optical transparency, excellent mechanical properties, and strong adhesion to various substrates. The synergistic covalent–noncovalent hybrid network enables efficient energy dissipation, and the CQAS-enhanced UCNP dispersion significantly improves the intensity and stability of conversion luminescence, as proven by the extended fluorescence lifetime. By employing distinct electrical and optical signal transmission pathways, the hydrogel serves as a highly sensitive resistive strain sensor for simultaneous mechanical and chemical monitoring, holding great promise for wearable electronics, smart healthcare, and environmental sensing systems.

To understand the potential of hydrogels and research directions for developing next-generation force sensors suitable for medical applications, we should first examine the differences between natural and synthetic hydrogels and the effects of their combinations on the physical and structural properties. The fundamental differences and technological limitations are presented in Table 1.

B. Wang [105] proposed a “mechano–nano-configuration synergy” strategy or quasi-static cold compression to fabricate poly(vinyl alcohol)/hydroxyapatite (PVA/HAp) nanocomposite hydrogels with alternating “hard–soft” interconnected architecture elements by exploiting certain temperature-induced transitions from viscous-elastoplastic to viscoelastic states, which allows for the mechanical incorporation of high-load hydroxyapatite reinforcements into the viscous-elastoplastic hydrogel, followed by structural stabilization by freezing-induced viscoelasticity. Implantable hydrogels (IHGs) occupy a unique position in surgery, as they enable dynamic tissue interactions, biodegradability, self-healing, and sustained drug release via surgical or minimally invasive approaches. The emergence of multifunctional, rapidly responsive IHG variants has further expanded their therapeutic, diagnostic, and regenerative potential while preserving the essential properties of the materials [106].

## 4. Nanomaterials for a Hydrogel Sensor

**Figure 3:**
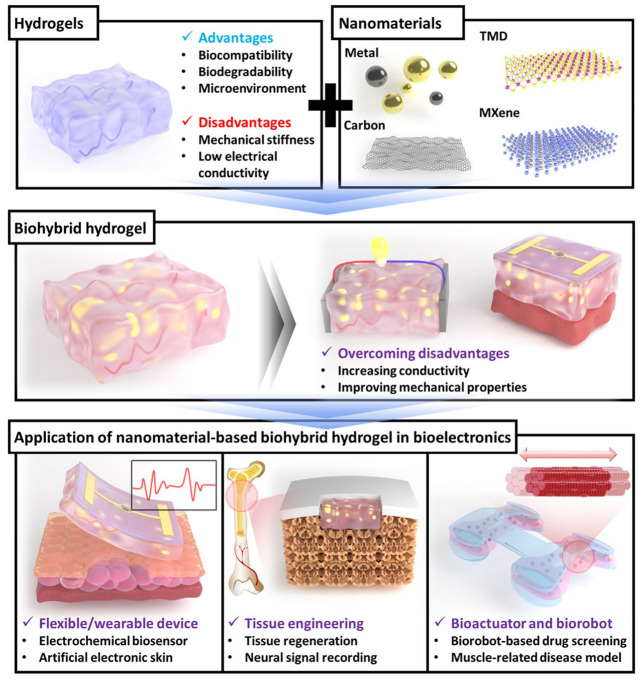
Graphical representation of hydrogel with nanomaterial application fields from ref. [107].

Although hydrogel bioelectronics have unique properties (Figure 3), there are still limitations, such as their low electrical conductivity and structural stability; nanomaterials can partially compensate for the shortcomings of hydrogels [107]. Nanomaterial-based hydrogels demonstrate great potential for wearable sensor fabrication, including with respect to the processing requirements. It is expected that continued research and development will lead to higher throughput, lower manufacturing costs, and more convenient monitoring of electrophysiological signals, opening up new perspectives for the development of wearable sensor technology [108]. The use of hydrogels for the production of sensors has a unique advantage, as they can transmit and modulate electrical signals by activating ionic or electronic conduction pathways, while maintaining dynamic interaction with the surface of biological tissues, which makes them suitable for use in both wearable electronics and implantable biological interfaces, and they can participate in the wound healing process, even in nerve regeneration or the regulation of cellular processes [64]. Hydrogels are generally not resistant to bacterial attacks, which degrades their stability. Therefore, the requirements for hydrogels are not only stability with a wide range of activity but also having antibacterial efficacy and antioxidant properties [109]. Polysaccharide-based nanomaterials and hydrogels have emerged as a promising class of biomaterials in the medical field. Polysaccharides make up the majority of these naturally occurring polymer families. Materials that spontaneously decompose into smaller, simpler components, such as sugars and gases, have specific requirements for proper use in sensor manufacturing. The N. Asthana [110] research examines synthetic biodegradable polymer-graphite oxide composites to evaluate their performance in sustainable electrochemical applications. For PVA to be fully biodegradable, specific bacteria and enzymes are necessary to facilitate degradation. PVA, or polyvinyl alcohol, is an artificial polymer that is also water-soluble. It is essentially made from polyvinyl acetate. The hydrolysis of polyvinyl acetate produces PVA, which is readily degraded by living organisms.

### Hydrogel Sensor with Carbon Nanomaterials

**Figure 4:**
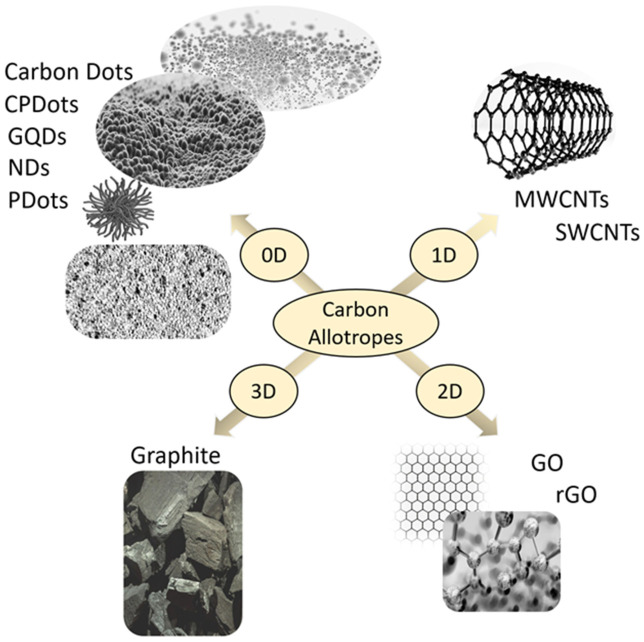
Visual representations of carbon allotropes are most commonly used to improve the hydrogel structure.

Nanostructured materials (Figure 4) with a large surface area are well-suited for developing conductive functional materials for electronics, and, when combined with hydrogels, they represent ideal material compositions for flexible electronics and wearable devices. Classical nanotechnologies have significantly transformed biomedical research; however, conventional nanoparticle synthesis often relies on toxic reagents, energy-intensive processes, and unsustainable practices that raise environmental and safety concerns [111]. Carbon-based nanomaterials are considered ideal conductive components to develop hydrogel-based materials due to chemical stability, biocompatibility, simple synthesis, and excellent electrical conductivity [112].

The use of graphene and CNT nanocomposites in hydrogels for sensors and their challenges, including slow response, is reported by C. S. Espenti [113]. The pH sensitivity refers to hydrogels made from ionogenic monomers or charge-forming groups, such as dimethyl-aminoethyl methacrylate, N-isopropylacrylamide, acrylic acid, methacrylic acid, poly(ethylene glycol), poly(2-hydroxyethyl methacrylate), copolymers of 3-trimethoxysilylpropyl methacrylate with poly(2-hydroxyethyl methacrylate), and polymers containing phosphoric acid derivatives.

End-of-life management of hydrogel products remains environmentally challenging. Hence, H. Xu [114] presented green-synthesized PVA/SA/CNTs-COOH hydrogel for human motion monitoring. The synergistic interaction of the PVA network, SA, and CNTs-COOH ensures consistent signal transmission and high sensitivity to deformation.

Nano-biotechnology is a growing body of biocompatible and environmentally sustainable nanomaterial technologies, also applied to the production of sensors. The so-called green synthesis has already become a promising alternative to conventional physical and chemical methods for producing nanoparticles. Excellent results are achieved by using/employing biological derivatives, such as plants, bacteria, fungi, algae, yeasts, and other microorganisms, as natural reducing and stabilizing agents, thus reducing the use of toxic chemicals and harsh reaction conditions [115].

Integration of electrically conductive materials into hydrogel matrices enables new capabilities in biomedical material manufacturing. Nevertheless, conventional methods of directly mixing components can compromise both functionality and biocompatibility. When discussing the use of carbon nanomaterials in hydrogels, we must emphasize not only the compatibility of devices based on these materials with living organisms but also the environmental sustainability of producing various carbon nanomaterials from renewable resources without fossil fuels. One source of carbon materials for biomedicals is biomass [116].

D. Chen [117] developed and reported a translucent, anisotropic CH composed of photo-crosslinking PVA-SbQ, sodium alginate, TA@CNC, with FeCl_3_ featuring a multi-system, oriented double-network structure, fabricated by a simple combination of UV crosslinking, pre-stretching for orientation, and secondary crosslinking in FeCl_3_ solution. This oriented architecture endows the hydrogel with strong adhesion, moderate transmittance, and outstanding anisotropy. Zh. Li [118] I was inspired by the principles of the Free-Form Reversible Embedding of Suspended Hydrogels (FRESH) printing technique, a method presented that encapsulates a patterned conductive material into high-water hydrogels via embedding printing when CNTs and pluronic acid are suspended in a polyvinyl alcohol hydrogel bearing styryl-pyridinium groups to encapsulate the conductive pattern.

The integration of nanomaterials into the hydrogel matrix enables tuning of properties to meet the specific sensing requirements. Nanomaterials can reinforce structure, enhance conductivity, create active functional sites, and overcome certain limitations of the hydrogel backbone. The graphene derivatives, such as GO and rGO, can improve electrical conductivity by providing a hybridized carbon network [119]. The GO is a 2D material with a honeycomb-like lattice composed solely of C atoms bonded via sp^2^ hybridization, which, when incorporated into hydrogels, improves the mechanical properties of the final product, thereby opening up applications in tissue engineering and drug delivery [120]. The graphene-based hydrogel was elaborated by A. Ahmad [121]. Gelatinization was achieved by stacking interactions between π–π bonds in GO reduced in aqueous solutions with hydrazine or ascorbic acid. Polymer dots represent a new class of sp^2^-rich, carbon-based nanomaterials. In comparison with carbon-based nanomaterials, polymer dots exhibit better biocompatibility, solubility, and surface functionalization capability, enabling the design of a visible light-responsive adhesive and a stretchable and conductive mineralized hydrogel with photothermal activity [122].

A composite hydrogel for sound detection presented in the Y.Wu [123] work, which uses PVA as the first network, polyacrylic acid (PAA) as the second network, and two-dimensional nanomaterials such as rGO as the conductive fillers, was developed in a single-step method and performs well in detecting physiological signals, including electrocardiograms, subtle vibrations, and body movements of various scales, highlighting its broad applications in biomedical and motion sensing fields. The uniformly distributed rGO in the hydrogel forms an efficient conductive network, giving the material high sensitivity (GF = 0.64), excellent conductivity (8.15 S m^−1^), fast response time (350 ms), and exceptional stability.

Solutions that utilize various wastes from other economic activities are economically attractive in the fight against waste. The E. Villegas [124] study used hydrothermally derived CQDs from avocado seeds and VCL-based thermosetting hydrogels with polyethylene glycol diacrylate (PEGDA) as a coupling agent. The combination of VCL-based hydrogels and carbon dots enhances photoluminescence compared to pure CQDs, making these materials potential biomedical thermo-responsive sensors.

Cellulose, especially cellulose nanocrystals, significantly improves mechanical properties and is very attractive to developers because it mimics the hierarchical arrangement and functional properties of living organisms in nature [125]. Cellulose hydrogels, formed by physically or chemically crosslinking cellulose or its derivatives into a three-dimensional network, are known for their exceptional water absorption capabilities and biocompatibility. The increasing demand for sustainable materials has stimulated interest in cellulose hydrogels due to their abundant supply, biodegradability, and non-toxicity [126]. The cellulose and graphene composite hydrogels represent a synergic combination of materials. Graphene exhibits high mechanical properties, thermal and electrical conductivity, and a large specific surface area, making it a promising carbon-based nanomaterial [127]. Nanocellulose (NC), isolated from natural cellulose resources and mainly composed of cellulose nanofibrils (CNFs) and cellulose nanocrystals (CNCs), has attracted increasing attention in recent decades due to its exceptional physicochemical properties [128].

H. Yang [129] developed eco-friendly CaTPD hydrogel low temperature polymerization initiated by a tannic acid–Ca^2+^ complex without an external energy source from cellulose biomass resources, resulting in a dual-crosslinked network with effective solving of leakage of inorganic hydrated salts, achieving high piezoelectric sensitivity. The elaborated hydrogel is suitable for sports, rehabilitation, pain management, and thermal therapy monitoring.

**Figure 5:**
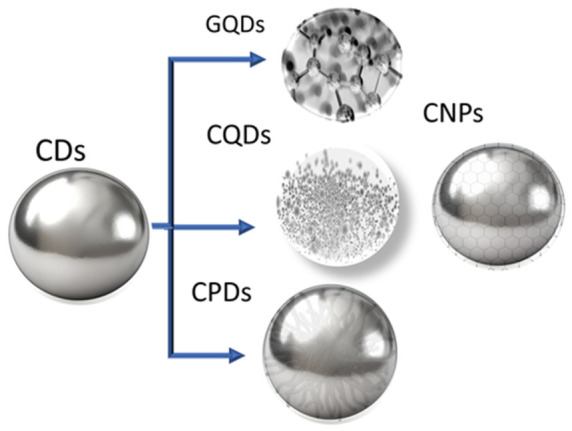
Classification of different types of carbon nanodots: CNDs, GQDs, CQDs, CPDs, and PDs.

Smart wound dressings that enable real-time sensor-based monitoring and personalized treatment are essential to advance personalized wound care. The bacterial cellulose, as a structurally strong, biocompatible material, has great potential for next-generation wound care technologies and for dressing-integrated biosensors [130]. Current, state-of-the-art sensor dressings lack sufficient wound repair capabilities and have only a single monitoring mode, which limits their clinical application, as Z. Yu [131] states. However, by using free radical polymerization of CMC, NIPAM, and Ga^3+^, a safe and simple dual-mode hydrogel sensor was developed for monitoring and therapy of infected wounds. In addition, the temperature/pH sensing functions enabled the hydrogel to monitor wound temperature and pH and to provide real-time alerts of wound infection based on changes in electrical signals. R. Xu presented another successful CH containing Ga^3+^ and suitable for sensor manufacturing [132]. The investigation of mussel-inspired PAM–PDA/Ga^3+^ integrated multifunctional hydrogel made by Ga^3+^ accelerated oxidative polymerization of dopamine resulted in PDA/Ga^3+^ NPs with excellent mechanical properties of the hydrogel and remarkable antibacterial properties against *Staphylococcus aureus* and *Escherichia coli*. The N. Nan [133] article presents a strategy for developing a multifunctional hydrogel patch by embedding carbon dots (C-dots) into an ionically crosslinked sodium alginate (SA) matrix, as shown in Figure 5. The C-dots play a dual role: they provide visible, pH-sensitive fluorescence for wound monitoring and act as superoxide dismutase (SOD)-like nano-enzymes to scavenge reactive oxygen species (ROS), with optical transparency and swelling capacity tunable by varying the number of C dots. The thermoelectric hydrogel elaborated for wound healing, biosensing, and energy harvesting is presented in the B. Tian [134] investigation, where ball-milled Mg_3_(Sb, Bi)_2_-based particles and CNTs play a key role to create a percolated ionic-electronic network providing high thermo-voltaic power.

Y. Wei [135] presented a multifunctional CH composed of CMC, PDA, and P-NPs for simultaneous movement monitoring, electromyography (EMG) signal detection, and photothermal therapy, synthesized through a one-step amidation/oxidative polymerization approach, yielding an interpenetrating dual-network architecture with enhanced mechanical strength and tissue adhesion relative to single-network systems.

The work of X.-Y. Du [136] presents a core-shell polymer/hydrogel microfiber strategy by integrating microfluidic spinning with shear-induced coating technology, where polycaprolactone (PCL) acting as a scaffold provides the tissues with structural and mechanical strength, and the hydrogel shell exhibits functional diversity, including high flexibility, self-healing, self-adhesion and pH-sensitive swelling ability, providing an excellent basis for application in personalized, smart and precise theranostic wound dressings. In addition, the tissue could be used as a flexible wearable sensor for real-time detection of human movements such as stretching, bending, and fist clenching.

However, it is difficult for conventional hydrogel strain sensors to simultaneously achieve high sensitivity, a wide strain range, and good self-healing performance; the attention on these materials has greatly increased. Biocompatible zwitterionic polymer hydrogels are considered ideal candidates for biomedicine, soft robotics, and wearable devices. MPC (2-methacryloyloxyethylphosphorylcholine)-based hydrogels are known to exhibit poor mechanical properties, and a new strategy to synthesize hydrogels with exceptional comprehensive mechanical properties based on multi-step crosslinking is proposed by Y. Yao [137], where with zwitterionic polymer hydrogel by adding a trace of chemical crosslinker to the synthetic system of PMDA-G hydrogel, a PMDA-GP hydrogel is obtained, which exhibits even better mechanical properties.

A separate group of hydrogels that often attracts the attention of researchers are graphene hydrogels, three-dimensional networks of graphene sheets impregnated with water, offering a versatile platform for the development of advanced materials with tailored properties. Graphene, known as the “one-atom-thick material in the universe” and containing sp^2^ hybridized carbon atoms, is a material that researchers are exploring for its potential applications. Due to its unique properties, such as electrical conductivity, large surface area, rich surface chemistry, and excellent electrical, thermal, mechanical, and optical properties, graphene is an exceptional material for a variety of applications, including electronics, energy storage, sensors, and medical devices [138]. Graphene in hydrogels significantly improves the mechanical properties, electrical conductivity, and thermal stability of sensors. The physical properties of graphene hydrogels can be easily adjusted by tailoring the functional groups to specific application requirements, and researchers can develop and produce new materials that can be applied in various fields, which is especially attractive for the development of healthcare devices [139].

The electromechanical, structural, and morphological properties of graphene-based hydrogels using various synthesis methods were analyzed by L. Mampane [140], with the aim of evaluating and revealing new ways to apply graphene-based hydrogels in healthcare. The successful example of an ionic cross-linked hydrogel-based multifunctional flexible sensor represented by T. Du [141] has good mechanical charge transfer properties, wide range linearity, and is manufactured without nanomaterial adds.

**Figure 6:**
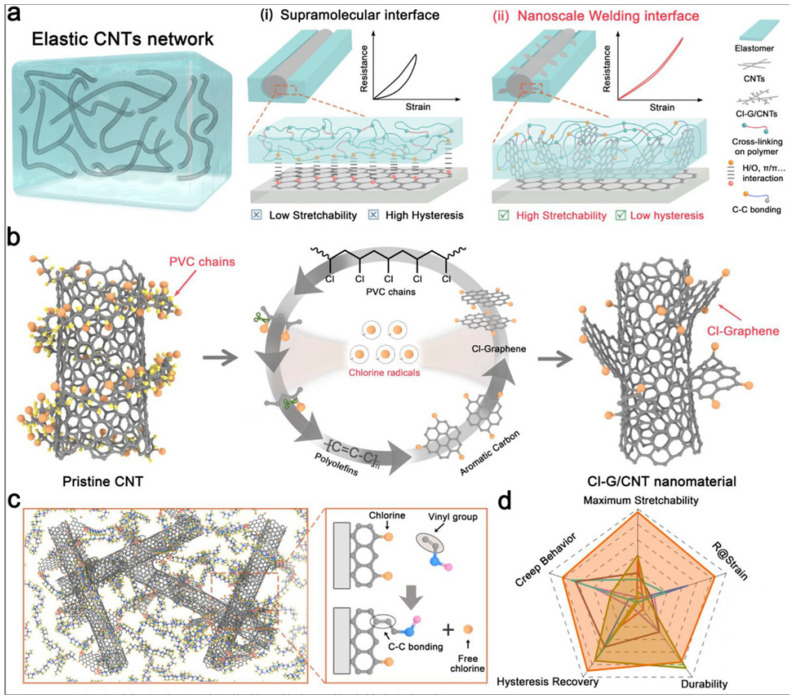
D. Ren [142] Conceptual illustration of in situ de-chlorination-triggered nano-welding strategy for low-hysteresis conductive elastomers. (**a**) Schematic illustration of interfacial binding strength and corresponding related electromechanical features for (i) commercial CNTs and (ii) Cl-G/CNTs nanocomposites. (**b**) Schematic fabrication for Cl-G/CNTs nanomaterials using PVC-assisted dehalogenation reaction. (**c**) Schematic illustration of in situ chlorine-releasing nano-welding strategy to construct robust covalent-bonding interface. (**d**) Comparison of the comprehensive electromechanical performance of this work and other CNTs-based conductive elastomers. “Reproduced with permission from [142], Ren© [2026] Wiley-VCH GmbH”.

D. Ren [142] presented a de-chlorination-triggered nano-welding strategy (Figure 6) for the improvement of CNT elastomers for strong chemical bonding and interfacial slip, a technology to create a 3D C─C covalent bond network between CNTs and silicone polymers. An elaborated sensor was validated in terrestrial and underwater environments.

Multifunctional sensors based on polymer hydrogels with carbon nanostructures, or on carbon-containing materials, are quite successful. The overview of multifunctional sensors is presented in Table 2.

With the addition of nanomaterials, hydrogels have achieved various excellent results in the period from 2023. Wearable physiological signal monitoring sensors made using nanomaterial-enhanced hydrogels have many advantages over traditional devices. As the biggest advantage compared with previous generations of sensors, these sensors not only fit perfectly on human skin without damaging it, ensuring accurate and stable monitoring of electrophysiological signals, but also can be used as wearable devices on various occasions and in difficult conditions [153].

M. Liu [144] elaborated the micropatterned hybrid sheet representing electrical conductivity by electron transport through the micropatterned nano-carbon aerogel, where deformation of the aerogel determines current change and partially carbonized BC retains the shape and holds the interconnected network inside the aerogel sheet. The manufactured sensor operates in a wide range of strain (0–1000% strain).

The self-healing, self-adhesive hydrogel-based sensor elaborated by J. Wang [145] with physical cross-links including π–π interactions working as a 3-dimensional force sensor represents multifunctional abilities as a sensor.

Examples of hydrogel-based sensors designed to measure a single parameter are given in Table 3.

Some of the publications not only discuss the possible uses of the hydrogel sensor they created but also emphasize the uniqueness of their work in using renewable resources, which is a highly desirable solution in modern industry. According to search data, about 1000 authors mentioned renewable resources in their works, of which 262 are research-type works in the period from 2023 to April 2026.

J. Li [169] presented a unique solution of the possibility to use seawater as natural antifreeze and a renewable resource for hydrogel with CNCs@HA and L-proline manufacturing. The produced hydrogel demonstrates rapid responsiveness (response and recovery times of 352.4 ms and 351.8 ms, respectively) and can be applied for low temperature medical devices. Here metal ions form a coordination–hydrogen bond dual network with PAA chains and CNCs@HA improving the toughness of the hydrogel. The corn starch hydrogel-based sensor was presented in the Q. Xu [170] work with high mechanical properties (tensile strain 1250.29%, modules and toughness of 1324.43 kPa, 9008.88 kJ m^−3^, respectively), and excellent anti-freezing (−128.9 °C), anti-bacterial, and electrical conductivity. A hydrogel-based sensor constructed from dopamine-encapsulated cellulose nanofibers (DA@CNF) and Cu^2+^, with high strain sensitivity (GF = 5.15) over a wide strain range (400%) presented in the S. Zong [171] work, emphasizes the use of cellulose as a renewable resource.

**Figure 7:**
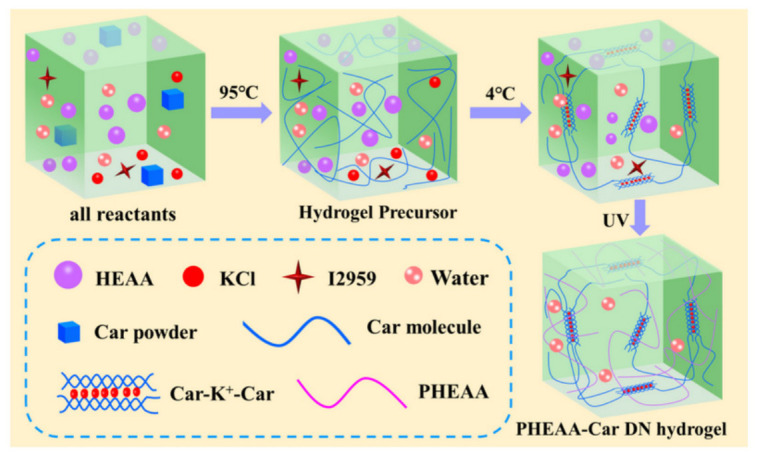
Preparation of PHEAA-Car hydrogel fabrication process by ref. [175].

The term “biodegradable” refers to the ability of a material to spontaneously break down into smaller components when exposed to microbes in the environment. A comprehensive review of biodegradable natural hydrogels is provided by L. Liu [172]. The zero-waste electronics trends are presented in the C. Jeyalakshmi [173] work. T. Kantasiri [174] presented CH from metal–phenolic networks and green tea elaborated for flexible electronics. Naturally biodegradable polymers, which are degraded by nature and organisms when released into the environment without causing major environmental damage, are called biopolymers. F. Ji [175] presented an acceptable for electronic skins and strain sensors conductive, transparent, self-healing, fully physically crosslinked double network hydrogel which was developed based on PHEAA and κ-carrageenan (Figure 7). For the physical gelation hydrogen bonds, ion bonding, and electrostatic interactions were employed.

Electrically conductive polymers have a very wide range of applications, being useful in various fields such as sensors, batteries, the petroleum industry, biosensors, biomedicine, catalysis, cancer treatment, etc. Electrochemical sensors based on conducting polymers have implications for neurochemical and pathogen detection, which are crucial neurochemical monitoring platforms for brain function and mental health [176].

**Figure 8:**
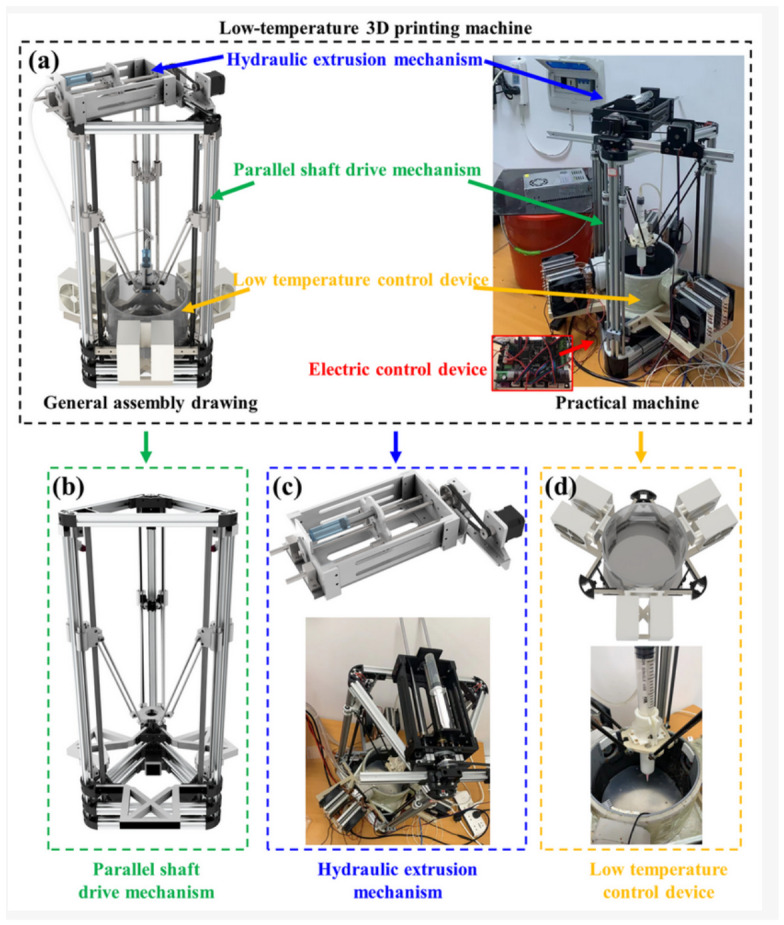
The (**a**) general assembly drawing and corresponding assembly drawings of (**b**) parallel-shaft drive mechanism, (**c**) hydraulic extrusion mechanism, and (**d**) low-temperature control device of the low-temperature 3D printing machine by Q. Zhao [180].

When discussing successful strategies for developing hydrogel-based sensors, it would be difficult to avoid 3D printing technology. This technology opens up possibilities for creating layered structures which, together with chemical crosslinking of molecules and other previously discussed technologies, allow the creation of hydrogel structures. 3D printing technology enables hydrogel-based sensors to be created with personalized design, a wide variety of geometry, and controlled microstructures. Microstructures made by 3D printing in hydrogel-based sensing material are not well characterized. A detailed understanding of how specific microstructural parameters determine the response characteristics of sensors would lead to more rational design approaches [177]. The S. Eckstein [178] study presents a generalized 3D printing system for continuous carbon fiber pseudo-woven composite mechanical metamaterials, called “meta-skins,” evaluates the processability and quality of the printed samples, and investigates their impact performance as a function of the interlacing pattern. These meta-skins were designed with repeating unit cells, additively fabricated using an automated fiber placement robotic printer. Sensitive and self-powered piezoelectric sensors, fabricated on PVDF for gait recognition, exploiting the unique properties of E-FDM 3D printing and CNT fillers, are presented in the work of K. Xu [179]. By adding CNT fillers and applying an external electric field during 3D printing, the amount of β-phase in PVDF can be significantly increased without secondary polarization, as the authors state. Sh. De [7] presented the evolution of strain sensor technologies from the 1990s—traditional bonded wire designs to a newest generation of flexible structures enabled by advanced materials and additive manufacturing using 3D printing technologies. This paper highlights breakthroughs in fabrication techniques, particularly fused filament fabrication (FFF), direct ink writing (DIW), and cubic photopolymerization (VPP), that address limitations of conventional methods, including complex multi-step processing and alignment challenges. The Q. Zhao [180] article on the low-temperature 3D printing technology (Figure 8), by using PVA matrix hydrogels, including PVA-LS hydrogel and PVA-CMC hydrogel, exhibited specific low-temperature rheological properties, laying the theoretical foundation for low-temperature 3D printing.

3D printing has revolutionized the development of flexible pressure sensors by enabling the precise fabrication of a variety of microstructures that significantly improve sensor performance. Zhen-Hua Tang [181], presented in his work, by combining conductive fillers (CNTs) and rheological modifiers (insulating microspheres, IHMS), a new class of printable three-component inks with suitable rheological properties, developed by 3D DIW printing of viscoelastic silicone-based inks consisting of CNTs and IHMs. The potential application of this representative printed pressure sensor for health monitoring was tested by the authors, and it successfully tracked and distinguished human movements, including walking and running. M. Li [182] presented a synthesized composite hydrogel (named SPBC) of poly(vinyl alcohol) (PVA)/sodium alginate (SA)/cellulose nanofibers (CNFs)/sodium borate tetrahydrate, which has good self-healing, electrical conductivity, and excellent mechanical properties. This SPBC0.3 hydrogel demonstrates rapid self-healing (<30 s) and achieves mechanical properties of 33.92 kPa, high tensile strain performance (4000%), electrical conductivity (0.62 S/cm) and electrical response properties, and available human motion monitoring, for 3D-printing personalized fabrication.

K. Zhang [183] presented a unique and valuable in vivo technology for manufacturing hydrogel sensors using near-infrared 3D printing, using core-shell conversion nanoparticles for direct photopolymerization, where the sensor printing directly on the mouse body can record locomotor movements and convert them into electrical signals in real time. This in vivo 3D printing method of flexible sensors, an excellent example of non-invasive technology, is designed for the individual production of hydrogel sensors, revealing the potential of hydrogels for personalized health monitoring and disease diagnosis. P. Zhang [183] responded to the needs of modern medicine for personalized care using near-infrared 3D printing, where the most striking feature of the represented sensor is in vivo one-piece body molding, facilitated by core-shell conversion nanoparticles combined with indocyanine green, which act as an embedded ultraviolet light source for direct photopolymerization. With this method, a sensor was printed directly into the mouse body and can record movements signals in real time. This in vivo 3D printing method of flexible sensors is considered a non-invasive methodology for the fabrication of individual hydrogel-based sensors, thus greatly promoting the potential of versatile hydrogels for individual health monitoring and disease diagnosis. The application of hydrogels by 3D and 4D printing technologies in the form of hydrogel inks holds quite promising prospects in personalized medicine for health status monitoring and for medical implants coatings [184] A 3D printed device by LCD-based vat photopolymerization of conductive photo-nanocomposite was presented by C.T. Chen [162].

The CNC hydrogels have emerged as promising candidates for a variety of biomedical applications due to their biocompatibility, sustainability, and adaptability [185]. The CNC as an additive for 3D printable inks in the D. Kumar [186] review discusses the understanding of the relationships between CNC surface chemistry, particle aspect ratio, and flow-induced particle alignment, as well as their relationship to the rheology of bio-ink printability and anisotropic bio-functionality suitable for biomedical sensor applications.

Conventional hydrogel sensors have difficulty in recovering their original function after damage, which limits their practical applications. The SPBC hydrogel, due to good rheological properties which support 3D printing methods, exhibits self-healing, good electrical conductivity, and good mechanical properties [183]. Successful sensor-embedded muscle incorporated in a biohybrid robot demonstrates closed-loop controllable actuation [187,188].

The 3D technology with machine learning and artificial intelligence control enables the baseline shift of hydrogel sensor to correct the nonlinear adaptive ability of the support vector machine; effective signals can be separated by using the local feature extraction of the convolutional neural network, and multi-parameter signal decoupling can be achieved by performing random forest feature importance analysis, thus laying the foundation for accurate data interpretation; long short-term memory networks can also be utilized to capture the dynamic correlation features of multi-sensor signals as Y. Zhang [12] states.

By summarizing recent advances and identifying opportunities for innovation, this review provides important insights on how to bridge the gap between research and real-world applications, helping to accelerate the evolution of flexible pressure sensors with complex 3D printed microstructures.

## 5. Conclusions and Future Trends

The period chosen for our study is quite short, so it is relevant to discuss not just one chosen direction of sensor development but to review the topics that have received the most publications. Based on the selected directions for sensor development (polymers and their combinations, carbon-containing nanomaterials, manufacturing technologies), we can draw the following conclusions on hydrogel-based sensor manufacturing. When evaluating statistical data, it should be noted that during the period under review, there were four times more review publications than scientific publications. We can conclude that scientific activity is currently producing less significant results and is looking for new methods and solutions. In the search for ways to develop cheap and health-friendly sensors, attention is usually drawn to renewable resources of biological origin and, at the same time, to hydrogel-based sensors for health monitoring. Degradation of conductive components limits the time during which devices can be reliably used, making such devices disposable or for personal monitoring where high accuracy is not required. Research is often aimed at creating stable hydrogels of natural origin that are resistant to degradation by using various molecular crosslinking techniques, crosslinking agents, and incorporating stabilizers or nanomaterials to ensure stable charge transfer.

Fabrication of hydrogel sensors often involves a trade-off between electrical conductivity and mechanical integrity. The future of hydrogel research in the field of medical applications is rapidly expanding, and several exciting directions are currently being observed. A review of recent publications identified a promising direction in hydrogel research, namely the development of multifunctional hydrogels that can respond to multiple stimuli simultaneously.

The topic of renewable and cheap resources is also relevant for sensor developers. The use of natural polymers as a renewable resource is most often emphasized, but there are also unique articles that see economic benefits even in the use of seawater. Obviously, the use of iron salts is a cost-effective and environmentally friendly solution.

After evaluating all the reviewed literature, it was found that hydrogel-based medical products used for wound monitoring are implanted or printed directly onto the surface of organs and are also intended for monitoring when robust adhesion to the skin is important. The CHs face the challenge of managing their stability and degradation over time. Although hydrogel-based structures initially exhibit impressive mechanical and electrical properties, environmental factors and biological fluids influence their structural integrity negatively. Using the science of material structure would be a detailed justification of how specific microstructural parameters determine the response characteristics of sensors, which would allow for more rational design approaches to modeling.

Successful hydrogel-based sensors fundamentally lack conductive nanomaterials with hydrogel compatibility that could optimize the permeation thresholds and interfacial adhesion to achieve balanced electromechanical properties in next-generation multifunctional sensors and, most importantly, maintain good compatibility with the skin surface and be body-friendly. Hydrogel-based flexible sensors face signal processing limitations where sweating and body temperature fluctuations might cause signal interference in human physiological signals and motion tracking applications.

Ionic hydrogels currently do not meet current expectations. To mimic human tissues in the field of personalized medicine, ionic hydrogel devices should have multifunctional sensing capabilities, simultaneously recording changes in temperature, pressure, humidity and chemical concentrations in high sensitivity, while demonstrating biomimetic properties, self-regulating hydration, adaptive stiffness, and autonomous physiological feedback mechanisms. Further development of CHs will likely focus on multifunctional design and multimodal sensing, incorporating biochemical signaling, mechanoresponsive elements, and immunomodulatory functions [189]. The need to create multifunctional devices is obvious and has a successful future. For example, the piezo-ionic hydrogels open up possibilities not only for low-voltage and high-capacity sweat-activated batteries, but also for the development of a piezoelectric hydrogel that can act as a force sensor.

By applying the latest modeling techniques to predict the behavior of hydrogels in biological systems, researchers can optimize hydrogel designs before synthesis, accelerating the development of new material combinations. This is particularly important when employing hydrogels of biological origin. Multifunctional hydrogels, with the ability to respond to combinations of physical, chemical, and biological signals, can realize complex drug delivery systems tailored to changing health status with response in a very short time frame, as they adapt to the dynamic unique and sometimes changing environment in the body. Low-temperature 3D printing technologies are an important step in developing a new generation of hydrogel sensors, which raises the need to improve 3D low-temperature printers and suitable hydrogels to achieve the ultimate goal of a more advanced sensor for health monitoring. Although 3D printing is a specific technology compared to the other sensor architecture development strategies we have discussed, this technology is exceptionally convenient for forming layers and thus creating a durable specific film structure.

The new trend in hydrogel application for biomedical issues is biohybrid robotics combining biological tissues and artificial systems resulting in muscle cell-based micro-bots and bio-robots. The understanding of the behavior of hydrogels continues to improve significantly, and the possibilities for creating fully autonomous hydrogel-based systems are growing with the increasing pace of scientific research. Judging by the growth in the number of publications in terms of citations, the future of hydrogel research promises rapid growth, although many innovations are in the preclinical stage. Publications that present the study, production, and application of a new hydrogel sensor to clinical trials are very scarce. The rapid development of flexible electronics requires high-performance CHs, but their end-of-life management remains challenging from an environmental perspective. Multi-dimensional data collected synchronously by multiple sensors, such as deformation, pressure, temperature, and pH value, often encounter problems such as noise interference, signal interference, and dynamic environmental fluctuations. This is an information niche that sensor developers could fill as they are best aware of the specifics of their product and the possible recycling paths. One of the topics that is often overlooked by manufacturers of hydrogel sensors is how the materials that make up the sensors will be recycled when the sensor becomes unnecessary for the user. Obviously, the issue of materials recycling is very complex, going beyond the basic activities of sensor developers.

In the articles we reviewed, we found practically no information about what solutions sensor developers offer to dispose of unnecessary and worn-out sensors so as not to increase the amount of non-recyclable waste.

Currently, the mechanism of 3D printed microarchitectures and hydrogel-based flexible sensor performance is still not sufficiently characterized, judging from the information gathered in the reviewed publications. The lack of quantitative structure–property relationships forces us to rely on detailed experimental data and make possible assumptions. Theoretical justification and detailed mathematical simulation would help find the optimal directions.

Therefore, the integration of deep machine learning and artificial intelligence technologies into 3D printed hydrogel flexible sensor systems will also become a mainstream direction to overcome various problems related to the hydrogel structure, while the obstacle of correct data reading is expected to help in the future. Thus, the main directions of possible improvement of hydrogel sensors are determined by the success of research by inventors from other scientific fields.

## Figures and Tables

**Table 1:** Comparison of polymer origin vs. hydrogel physical/mechanical properties.

Hydrogel Type	Performance	Advantages	Limitations	Ref.
Natural origin	Low mechanical strength; Limited capacity; Ionic/covalent crosslinking;	Biodegradability; Biocompatibility	Fast degradation; Weak mechanical strength	[98,99]
Synthetic	Easy engineering; Good stiffness; durability	Durability; Repeatability; Customizable properties;	Toxicity of manufacturing bioactivity; poor adhesion	[100,101]
Hybrid: natural and synthetic	Improved strength; stiffness	Bioactivity; Biocompatibility; Reinforcement of structure; Combined synergic features of bioactivity with strength	Higher cost; Challenges in repeatability	[102,103,104]

**Table 2:** Overview of multifunctional sensors based on polymers with carbon nanomaterial.

Polymer	Nanomaterial	Parameters	Ref.
PAAm/PVA/TA@AL- +Fe^3+^	Alkaline lignin nanoparticles	tensile strength of 115 kPa; strain of ∼900%; toughness of 0.45 MJ m^−3^; ionic conductivity = 0.75 S m^−3^	[70]
A_1_-P_6_-PDMS	MWCNTs	GF = 27.63; elongation at break >140%; tensile strength >2.5 MPa	[143]
nano-carbon aerogel/BC-P(AM-SMA)-Al^3+^	BC, nanocarbon	GF = 12.3ε^1.27^; GF = 5.10 50 % strain; GF = 229.1 at 1000 % strain; S = 31.33p^−0.71^; response = 83.3 ms; cycling stability over 1000 cycles;	[144]
P (AA-HEMA)/PDA@CNT	PDA@CNT	GF = 5.46; Compressive sensitivity = 53.13 kPa^−1^; piezoresistive performance = 54.18 kPa^−1^; stability over 2000 cycles;	[145]
PDA (P(AA-HEMA)/St/rGO	GO, rGO	GF = 5.1; 176 kPa tensile stress; 1258% strain; conductivity (from 3.82 to 3.64 S/m); heating rate of 0.8 °C/s;	[146]
PAM	Cellulose nanofibrils	GF = 13.66; stress = 0.75 MPa, strain = 1593%; conductivity = 2.29 S/m;	[147]
PAM, MCNF	Cellulose nanofibers	tensile strength = 116.86–277.84 kPa, strain = 1923–2029%, and toughness = 1.34–2.12 MJ m^−3^;	[148]
PVA, (PVA-Borax-GaInSn-MXene)	MXene nanosheets	GF = 5.32; toughness = 1350.2 kJ/m^3^; strain range = 617.46%; recovering ~580% strain; response time = 170 ms; adhesion = 75.6 kPa (peak stress)	[149]
PEI	GO	GF = 2.26; conductivity = 0.46 S/m; adhesion = 13.7 kPa; stress: 113.5 kPa; strain: 1672%	[150]
Starch, amylopectin, PVA	MXene	GF = 1.1; stretchability ∼6151%; sensing range up to 300%;	[151]
PAM/HACC	OH-MWCNTs; CNT = 0.02 wt%	GF = 16.04; conductivity = 0.31 S/m; tensile strength = 435 kPa; elongation at break = 2500%;.	[152]
PVA/P(AA-NIPAM)-Fe^3+^	Ionic cross-linking	GF = 3.77; elongation = 475 ± 27%; tensile strength = 1.91 ± 0.05 MPa	[141]

**Table 3:** Samples of hydrogel-based sensors manufactured for single parameter sensing.

Function	Polymer	Nanomaterial	Parameters of Sensor	Ref.
Vibration	PVA, PDA	rGO, GO	GF = 0.64; conductivity = 8.15 S m^−1^; response time = 350 ms; hydrogel’s adhesion = 0.89 kPa	[123]
Strain	PANI/3-ABSA-ENR	ENR nanospheres	GF = 21.6; tensile strength 10 MPa; response time = 230 ms;	[154]
Motion monitoring	BC + AM	BC-confined MXene	GF = 11.48 at 600–800%; mechanical resilience = 6.5 MJ/m^3^ Elongation ~1800%; conductivity = 435.6 mS m^−1^	[103]
Force monitoring rehabilitation training	TPU	MWCNTs	GF = 10.065 0–3% strain range; GF = 5.419 in the 17–38%; GF = 0.219 at 38–62.80%; detection range = 0.039–2200 kPa; sensitivity = 0.029–9.912 kPa^−1^; recovery time: 50 ms–66 ms; durability 3735 cycles;	[155]
Motion sensor	PVA	PCM with MWCNTs	GF = 5.72; Sel = 13.2 mS/cm; durability over 500 cycles;	[156]
strain	PAA	CNCs/MWCNTs	GF = 7.07; tensile strength from 0.159 to 0.180 MPa; self-healing efficiency at 88.33%; swelling ratio = 39%	[157]
wearable epidermal sensor	chitosan	polydopamine-coated Mxene	GF = 1.73; specific capacitance = 373.41 mF/cm^2^, energy density = 74.67 μWh/cm^2^, capacitance retention = 82.43%; 5000 cycles,	[158]
human movement	PDMS/silicone rubber layers	Mxene/GO	sensitivity = 51.36; response time = 60 ms; strain detection lower limit of 0.1%	[159]
tensile	κ-Car/PAA/	GO	strength, toughness, flexibility (σf = 0.370 vs. 0.075 Mpa, εf = 1361 vs. 602%, E = 0.069 vs. 0.015 Mpa, W = 2.58 vs. 0.21 MJ/m^3^,	[160]
Strain sensor	CDs/CL/PVA	Nano carbon dots	GF = 5.34; toughness = 18.83 MJ/m^3^; tensile strength of 7.5 Mpa; conductivity = 24.5 mS/m; break of 487%; 1000 s cycle	[161]
piezoresistive device, LCD	Photo-resin	MWCNT	resistivity of ∼7.2–7.7 Ω; GF = ∼1.1–1.2; TCR ∼−1.58%K^−1^ for zero strain at 300 K; ΔR/R0, of ∼9.6–10.4;	[162]
Motion/sweat	PVA	rGO-APBA	self-healing capability >89%; sensing detection range = 0–550%; detection limit = 0.96 μM; mechanical = 2.23 MPa	[163]
Resistive sensor for breathing pattern	PEDOT:PSS/PVA/SA	PVA crystalline peaks; liquid metal nanoparticles; carbon-based nanomaterials	electrical conductivity = 48.69 mS m^−1^; fracture strength = 45.38 kPa, ductility = 209.13%; detect small deformation (2.5%); temperature response = −2.43 Ω K^−1^, 7.91 mV K^−1^;	[164]
heart rate and pulse monitoring	poly (acrylic acid)-TA@CNF (PTCCG-Na^+^) hydrogel	CNTs	GF = 4.47; resistance = 2.82 Ω; capacitance: 422.545 mFcm^−2^; environmental tolerance = −40–80 °C; stability >2000 cycles conductivity = 72.88 mS cm^−1^	[165]
real-time physiological monitoring and infection detection	alignate–gelatin ink	MXene vs. AuNP	Conductivity S = 0.44 S/m for AuNP-modified; S = 1.04 S/m for MXene; sensitivities in the range from −1.54–2.00% °C^−1^	[166]
human movements in extreme environments	polyvinyl alcohol–guar gum	cellulose nanocrystals 99.0%, (156.02)n g/mol	GF = 2.72; tensile modulus = 2.11 MPa; 92.94% self-healing	[167]
Resistive strain sensor	PNIPAM/PPy//PAA-Fe^3+^/clay	PPy nanoparticles	electrical conductivity = 1.24 ± 0.04 S/m; GF = 3.44;	[168]

## Data Availability

The raw data supporting the conclusions of this article will be made available by the authors on request.

## Abbreviations

AA	acrylic acid
ANF	an aramid fiber
AgNWs	silver nanowires
AM	acrylamide
BC	bacterial cellulose
CaTPD	tannic acid–Ca^2+^ complex + polyacrylamide
CDs	coal-based carbon quantum dots
CH	conductive hydrogel
CL	carbonized lignin
CMC	carboxymethylcellulose
CNC	cellulose nanocrystal
CNCs@HA	hyaluronic acid (HA)-functional cellulose nanocrystals
CNN-LSTM	convolutional neural network-long short-term memory
CNT	carbon nanotubes
CPDs	carbonized polymer dots
CB[6]	cucurbit[6]uril-modified
CQDs	carbon quantum dots
DEMA	N,N-diethylacrylamide
DMDAAC	dimethyl diallyl ammonium chloride
EMI	electromagnetic interference
f-CNTs	functionalized carbon nanotubes
FRESH	the Free-Form Reversible Embedding of Suspended Hydrogels
GQDs	graphene quantum dots
GO	graphene oxide
HEC-Ac	hydroxyethylcellulose with citric acid
HEMA	hydroxyethyl methacrylate
LCD	liquid crystal display
κ-Car	κ-carrageenan single-network hydrogel
IPN	Interpenetrating polymer networks
MCNF	matrix, dual-cellulose hierarchical network
MD	molecular dynamics
ML	machine learning
NC	nanocellulose
NIPAM	N-isopropylacrylamide
NPs	nanoparticles
NDs	nano-diamonds
OH-MWCNTs	hydroxylated multi-walled carbon nanotubes
PAA	polyacrylic acid
PAA/CNCs/MWCNTs	polyacrylic acid/cellulose nanocrystals/multi-walled carbon nanotubes
PAAm/PVA	polyacrylamide/polyvinyl alcohol
PAM	polyacrylamide
PAM–PDA/Ga^3+^	polyacrylamide–polydopamine/gallium(iii)
PAM/SA	polyacrylamide/sodium alginate
PAM/HACC	polyacrylamide/quaternary ammonium chitosan
PANI/3-ABSA-ENR	polyaniline/3-aminobenzenesulfonic acid-epoxidized natural rubber
PCM	multifunctional poly (vinyl alcohol)/chitosan/multi-walled carbon nanotube
PDMA	poly(N,N-dimethylacrylamide)
Pdots	polymer dots
PDA	polydopamine
PDA@CNT	polydopamine-encapsulated carbon nanotubes
PDMS	polydimethylsiloxane
PEI	polyethyleneimine
P(AA-HEMA)/St/rGO	poly (acrylic acid-2-hydroxyethyl methacrylate)/starch/reduced graphene oxide hydrogel
PHEAA	poly(hydroxyethyl acrylamide)
PNIPAM	poly(N-isopropylacrylamide)
P-NPs	polypyrrole-polydopamine-MnOx nanoparticles
PSCLE	PAM/SA/CNTs/LM/ethylene glycol hydrogel
PPy	polypyrrole
PVA	polyvinyl alcohol
PVA-CMC	PVA-sodium carboxymethylcellulose
PVA-SbQ	styryl-pyridinium-functionalized polyvinyl alcohol
PVA/HAp	poly(vinyl alcohol)/hydroxyapatite
PVA-LS	PVA-sodium lignosulfonate
PVA/SA	polyvinyl alcohol/sodium alginate
PVA/P(AA-NIPAM)-Fe^3+^	polyvinyl alcohol/poly(acrylic acid-N-isopropyl acrylamide)-Fe^3+^
PRGO	partially reduced graphene oxide
S	pressure sensitivity
SMA	stearyl methacrylate
SPBC	poly(vinyl alcohol) (PVA)/sodium alginate (SA)/cellulose nanofibers (CNFs)/sodium borate tetrahydrate
rGO	reduced graphene oxide
rGO-APBA	reduced graphene oxide-3-Aminophenylboronic acid
SA	sodium alginate
TA@AL	tannic acid-modified lignin
TA@CNF	tannic acid-encapsulated cellulose nanofibers
TA@CNC	tannic acid-coated nanocrystalline cellulose
TCR	temperature coefficient of resistance
VCL	vinylcaprolactam

## References

[1] Kulkarni M.B., Rajagopal S., Prieto-Simón B., Pogue B.W. (2024). Recent advances in smart wearable sensors for continuous human health monitoring. Talanta.

[2] Li J., Fang Z., Wei D., Liu Y. (2024). Flexible Pressure, Humidity, and Temperature Sensors for Human Health Monitoring. Adv. Healthc. Mater..

[3] Sengar R., Dubey S. (2026). Introduction and History of Hydrogels. Applications of Hydrogels in Modern Wastewater Treatment.

[4] Radha P., Nirubarani J., Sayyed N., Padmanabhan S., Priya A. (2025). Interdisciplinary Collaboration: Health IT Meets 6G. Patient-Centric 6G: A New Era in Smart Healthcare.

[5] Mihailidis A. (2026). Interdisciplinary Collaboration: Bridging Gaps to Advance AgeTech Innovation. The Age of AgeTech.

[6] Bathaei M.J., Bathaei Y., Liao Z., Yazdanmehr M., Sethi S.S., Nikolayev D., Cardoso F.A., Bounty C.M. (2026). Environmental and Ecological Monitoring with Biodegradable Technologies. Adv. Sci..

[7] De S., Xu S., Tang Y., Jackson A., Wang P.L., Rukmani S.J., MacDonald E., Zhao X., Duty C., Roschli A. (2026). Printing the future of strain measurement: Flexible sensors via additive manufacturing for wearables, robotics, and smart infrastructure. Sens. Int..

[8] Dube A., Malode S.J., Alodhayb A.N., Mondal K., Shetti N.P. (2025). Conducting polymer-based electrochemical sensors: Progress, challenges, and future perspectives. Talanta Open.

[9] Zheng H., Pochan D.J. (2025). Spiers Memorial Lecture: Recent advances (and challenges) in supramolecular gels. Faraday Discuss..

[10] Mo X., Wang H., Luo B., Zhang J., Cai C. (2026). High-Performance Hydrogels for Triboelectric Tactile Sensing: Principles, Design, and Applications. Adv. Funct. Mater..

[11] Wang Y., Gao Y., Tang L., Guo Y., Sha B., Jiang Y. (2026). Hydrogel-based wearable and implantable biosensors in health monitoring. Biomater. Sci..

[12] Zhang Y., Li X., Wu W., Hong W., Jiao T. (2026). 3D Printed Hydrogel Flexible Sensors: Fabrication Techniques, Sensing Mechanisms, and Application Advances. ACS Appl. Polym. Mater..

[13] Jing X., Xu Y., Zeng J., Feng P., Li S., Mi H.Y. (2026). Gradient-Structured Hydrogel with Integrated Actuation and Strain Sensing for Biomimetic Robotics. Adv. Funct. Mater..

[14] Gu Y., Luo Y., Guo Q., Yu W., Li P., Wang X., Ye T., Chang H., Yuan W., Wu H. (2026). Empowering Human-Machine Interfaces: Self-Powered Hydrogel Sensors for Flexible and Intelligent Systems. Adv. Funct. Mater..

[15] Shi W., Feng Z., Zhu X., Wang L. (2026). Fully Self-Powered Multimodal Flexible Sensors: Multi-Mechanism Energy Conversion for Next-Generation Wearable Electronics. Adv. Sustain. Syst..

[16] Chen R., Guo R., Li Y., Yan H., Huang J., Xu H., Zhang Z., Zhuo S., Liu M. (2026). Hydrogel Ionic Sensory Systems. Mater. Chem. Front..

[17] Zhi C., Wang C., Wu H., Guo C., Zhou X., Shi S., Si Y., Hu J. (2026). Self-powered functional hydrogel bioelectronics: From material design to biomedical applications. Matter.

[18] Mo F., Lin Y., Liu Y., Zhou P., Yang J., Ji Z., Wang Y. (2025). Advances in ionic conductive hydrogels for skin sensor applications. Mater. Sci. Eng. R Rep..

[19] Li N., Luo X., Zhu H., Shen G., Li M., Zhou C., Liang N., Zhang P., Zhu X. (2026). Hydrogel composite nanomaterials for diabetic wound treatment. Colloids Surf. B Biointerfaces.

[20] Cao X., Zhang T., Zha B., Li S., Huo F. (2026). Nanoparticle-enhanced functional hydrogels: From design, characterization to their biomedical applications. Eur. Polym. J..

[21] Yang H., Lu H., Mialo Y., Cong Y., Ke Y., Wang J., Yang H., Fu J. (2022). Non-swelling, super-tough, self-healing, and multi-responsive hydrogels based on micellar crosslinking for smart switch and shape memory. Chem. Eng. J..

[22] Tian Y., Li Y., Xu M., Li W., Cong H., Yu B. (2026). Polyvinyl alcohol Hydrogels: Structure, Preparation, Modification Strategies and Biomedical Applications. Eur. Polym. J..

[23] Chen Y., Lv S., Liu L., He T., Xu J., Liu J., Liu X., Mu Y., She J. (2026). A multifunctional ion-conductive hydrogel with supramolecular interactions for wearable sensing: Human motion monitoring and Morse code communication. Chem. Eng. J..

[24] Sun Q., Yao J., Wang L., Liu W. (2026). A gum Arabic-augmented hydrogel with high toughness, robust adhesion, and antibacterial efficacy for motion sensing and rehabilitation training. Talanta.

[25] Xue Y., Ni Z., Wang Y., Shan L., Liu J. (2026). Engineering Conducting Polymer Hydrogels for Bioelectronic Interfacing. Adv. Funct. Mater..

[26] Li D., Zhou H., Liu L., Jing C., Ji K., Liu G., Bai H., Yuan H., Wei H., Zhang W. (2026). Dual-Anchored Interfacial Adhesion-Enhanced Strain-Insensitive Hydrogels Electronic Skin for Consciousness-Driven Brain-Computer Interface. Adv. Funct. Mater..

[27] Lee K., Gul O., Kwon Y., Jeong J., Cho S., Ahn J., Yu J., Kim C., Lee D., Han H. (2026). Smarter Sensors Through Machine Learning: Historical Insights and Emerging Trends across Sensor Technologies. Adv. Funct. Mater..

[28] Liu S., Zhan H., Wu P., Lei Z. (2026). Underwater-Stable Conductive Hydrogels: From Molecular Design to Next-Generation Aquatic Sensors†. Chin. J. Chem..

[29] Theyagarajan K., Saikrithika S., Kim Y.J. (2026). MXene- and MOF-Based Hydrogels: Emerging Platforms for Electrochemical Biosensing and Health Monitoring. Micromachines.

[30] Wang Q., Gul O., Kwon Y., Jeong J., Cho S., Ahn J., Yu J., Kim C., Lee D., Han H. (2026). Robust, highly conductive, and recyclable hydrogel with tunable sensitivity for advanced flexible electronics. Mater. Today.

[31] Chang Y., Jia Y., Pan Y., Wang J., Yang H., Zu M., Cheng H. (2026). Enhancing water retention in hydrogels under extreme conditions: Strategies, applications and challenges. Mater. Sci. Eng. R Rep..

[32] Li Y., Liu R., Li Y., Chen G., Wan Y., Tian Y. (2026). PVA-based composite hydrogels for biomedical applications. J. Mater. Chem. C Mater..

[33] Haghighat H.R., Naqvi M., Moghari S., Naderi G., Khonakdar H.A. (2026). Conductive Hydrogels as Next-Generation Materials for Flexible and Wearable Electronic Systems. Polym. Eng. Sci..

[34] Kang X., Wang Y., Chen C., Liu J., Zhai X., Niu C., He X., Chen X., Li J. (2026). MXene-based multi-component conductive hydrogel with synergistic crosslinking networks for high-performance wearable sensors. Nanoscale.

[35] Du Y., Qin G., Han C., Rong Y., Du H., Wang Z. (2026). PEG-based conductive gels with ‘ring-wrapped chains’ dynamic crosslinking used for flexible wearable devices. J. Mater. Sci..

[36] Wu R., Zhu T., Ni Y., Wu C., Wang W., Zhao K., Huang J., Lai Y. (2026). UV-Cured Dense Double Network Hydrogel via Multiple Dynamic Crosslinking for Stable Amphibious Motion Sensing. Adv. Funct. Mater..

[37] Lou M., Ma Z., Shi L., Zhang J., Li W., Wang H., Jia L. (2026). Confined-space volume compression for ultra-thin and tough composite hydrogel films via enhanced hydrogen bond crosslinking. Chem. Eng. J..

[38] Zar Y., Htwe N., Pawłowska S., Jaafar M., Htwe Y.Z.N., Pawłowska S. (2026). Emerging Strategies for the Fabrication of Conductive Hydrogels from Conductive Polymers and Their Composites for Wearable Sensors, Energy Storage, and Biosensor Applications: Methods, Mechanisms, and Future Perspectives. Adv. Mater. Technol..

[39] Tordi P., Tamayo A., Jeong Y., Han B., Kayal B.-A., Cavallo A., Bonini M., Samori P. (2026). Fully Bio-Based Gelatin Organohydrogels via Enzymatic Crosslinking for Sustainable Soft Strain and Temperature Sensing. Adv. Funct. Mater..

[40] Liu C., Huang H., Yu X., Luo Y., Jianh Y., Li Y., Zhang X., Wan Z., Ji J., Yu Y. (2026). Machine Learning-Validated Silk Fibroin Composite Hydrogels: Robust, Antifreezing, and Conductive Sensors. ACS Appl. Polym. Mater..

[41] Zheng T., Cheng C., Wang D., Cheng C.C. (2026). Electronic jelly: Engineering the mechanics of hydrogels for flexible electronics. FlexMat.

[42] Ramkumar V., Rukmanikrishnan B., Endla P., Kim S.C., Shanmugan S., Madhappan S. (2026). Recent advances in the smart biopolymer nanocomposite hydrogels for biomedical interfaces and therapeutics. J. Mol. Liq..

[43] Barhoum A., Sadak O., Ramirez I.A., Iverson N. (2023). Stimuli-bioresponsive hydrogels as new generation materials for implantable, wearable, and disposable biosensors for medical diagnostics: Principles, opportunities, and challenges. Adv. Colloid Interface Sci..

[44] Farahbakhsh J., Najafi M., Al-Shaeli M., Benkhaya S., Zargar M., Iglauer S., Vatanpour V. (2026). Next-generation thermo-responsive materials: From hydrogels for biomedical applications to smart membranes for separation technologies. Sci. Total Environ..

[45] Ramesh A., Subbarayan R., Srinivasan D., Balakrishnan R., Shrestha R., Chauhan A. (2026). Matrix-Based Hydrogels in Biomedical Applications: Design, Functionality, and Translational Insights. Biomed. Res. Ther..

[46] Bisht N., Yeo R.J., Ramakrishna S., Sankaranarayanan S.K.R.S., Dhand C., Dwivedi N. (2026). Shape Memorable and Self-Healable Smart Hydrogels and Emerging Directions. Adv. Healthc. Mater..

[47] Pande S., Sahu I., Pati F., Chakraborty P. (2026). Merging Supramolecular Chemistry with Nanotechnology for Hydrogel-Based Soft Electronics. ChemNanoMat.

[48] Mascini M., Palmieri S., Eugelio F., Rivero M.I., Del Carlo M. (2026). Analytical Performances of Polymer-Based Biosensors for Real Samples Application. Biosensors.

[49] Song Y., Yang M., Liu T., Sun C., Jin M., Tan H., Zheng D., Zhang Y. (2026). Design of a flexible cellulose framework via supramolecular structure modulation and its application in hydrogel sensors. Carbohydr. Polym..

[50] Zeng Y., Li X., Tu Y., Zeng Y., Qiu L., Liu H., Yu J. (2025). Robust supramolecular conductive hydrogels based on silk fibroin and wool keratin for stretchable, self-healing, and adhesive strain sensors. Chem. Eng. J..

[51] Chen X., Tian P., Zhang T., Wang Y., Guo H., Li Q., Ti B. (2025). Soft/hard double network supramolecular hydrogel with multifunctional properties for flexible strain sensor. Colloids Surf. A Physicochem. Eng. Asp..

[52] Tang Z., Yang J., Li S., Wu Z., Mondal A.K. (2024). Anti-swellable, stretchable, self-healable, shape-memory and supramolecular conductive TA-based hydrogels for amphibious motion sensors. Eur. Polym. J..

[53] Lei T., Duan X., Zhalo H., Ma S., Ma X., Wang N., Zhang Q., Wan A., Xia Z., Shou W. (2025). A multifunctional flexible wearable hydrogel sensor with anti-swelling via supramolecular interactions for underwater motion detection and information transmission. Chem. Eng. J..

[54] Xia F., Huang Y., Yang Y., Chen H., Xu C., Liu S., Yang Q., Zhang X. (2026). Chitosan-mediated supramolecular hydrogel strengthened by freezing and salting-out showing self-healing, stretchable and biocompatible for flexible electronic. Polymer.

[55] Li S., Wang X., Du L., Li Y., Tang Z., Hu J., Zhang W. (2025). Supramolecular hydrogel membranes reinforced with aramid nanofibers via the dynamic cross-linking and hydrogen bonding network. Chem. Eng. J..

[56] Fang D., Bian J., Xiang Y., Wang Y., Zhu T., Kang Y. (2025). Self-Assembled Zwitterionic Lignin-Induced Joint Enhancement of Mechanics and Adhesion in High-Performance Hydrogels for Flexible Strain Sensors. Biomacromolecules.

[57] Yang Y., Huang Y., Chen H., Chen L., Liu S., Zhang X. (2024). Supramolecular hydrogels mediated by cucurbit[6]uril-modified Fe_3_O_4_ with self-healing, photothermal responsiveness and stretchability for flexible electronics. Colloids Surf. A Physicochem. Eng. Asp..

[58] Wang L., Wei J., You M., Jin Y., Li D., Xu Z., Yu A., Li J., Chen C. (2025). Initiatorless polymerization of mechanically robust hydrogels reinforced by cellulose of wood skeleton as multifunctional sensors. Carbohydr. Polym..

[59] Lin Z., Li G.-Z., Tao L. (2026). Development of self-healing hydrogels with new functions using nanomaterials. Nano Res..

[60] Adnan N.A., Bakar N.A., Zahid N.I., Nordin N. (2025). Emerging nanomaterials for conductive electroactive and stretchable hydrogels in biomedical microdevices. Polymer.

[61] Zhang X., Chen X., Ye Z., Liu W., Liu X., Wang X. (2023). Conductive hydrogels for bioelectronics: Molecular structures, design principles, and operation mechanisms. J. Mater. Chem. C Mater..

[62] Edo G.I., Nwachukwu S.C., Gaaz T.S., Emumejaye K., Iwanegbe I., Oghroro E.E.A., Owheruo O., Yousif E., Jikah A.N., Igbuku U.A. (2025). Smart biopolymers in commercial practice: A review of applications and challenges. Polym. Bull..

[63] Beg M., Sam V., Kanathedath J., Markapudi P.R., Alcock K.M., Goh K., Yu H., Manjakkal L. (2026). Multifunctional ultra-low voltage sweat-activated battery using piezo-ionic hydrogel. Nano Energy.

[64] Song B., Zhang J., Hao S., Shao C., Fu P., Wen J., Yang J., Cong H., Pan C. (2026). Multifunctional Hydrogel Interfaces: Reshaping the Future of Flexible Electronics. Adv. Mater..

[65] Choudhury S., Wang Z.L., Kim S.W. (2026). Hydrogel-based piezoelectric materials and devices for implantable bioelectronics. Biomaterials.

[66] Chen Y., Guo X., Zhang Y., Yang Z., Meng J., Li P., Ni Y., Huang Z., Wu H., Wei Q. (2026). Recent advances in plant polyphenol-based adhesive hydrogels for biomedical applications. Biomater. Adv..

[67] Sun Y., Tian G., Deng W., Yang W. (2026). Ionic Hydrogel Sensors toward Next-Gen Personalized Healthcare. Adv. Mater..

[68] Wu J., Hong J., Gao X., Wang Y., Wang W., Zhang H., Park J., Shi W., Guo W. (2025). Recent Progress in Flexible Wearable Sensors Utilizing Conductive Hydrogels for Sports Applications: Characteristics, Mechanisms, and Modification Strategies. Gels.

[69] Zeng L., Li Z., Wu D., Lan X., Xu S., Zhang Y., Dong J., Zhang D., Han S., Huo P. (2026). Injectable dual-network conductive antimicrobial hydrogels from oxidized dextran/hydroxypropyl chitosan: Multifunctional applications in wound healing and physiological monitoring. Int. J. Biol. Macromol..

[70] Li Y., Wang Y., Qiao Y., Liu X., Wang H., Zhang S., Yin X., Wang X., Han P., Gu Z. (2026). High-performance lignin-based hydrogel strain sensors for human motion monitoring. J. Colloid Interface Sci..

[71] Lee C., Huang H.-S., Wang Y.-Y., Zhang Y.s., Chakravarthy R.D., Yeh M.-Y., Lin H.-C., Wei J. (2023). Stretchable, Adhesive, and Biocompatible Hydrogel Based on Iron–Dopamine Complexes. Polymers.

[72] Guan X., Ma X., Gao R., Zheng Q., Sun C., Chen Y., Mu J. (2026). Low-Temperature One-Pot Fabrication of a Dual-Ion Conductive Hydrogel for Biological Monitoring. Sensors.

[73] Ren J., Wang Y., Liu Z., Liu K., Xiang X. (2024). Balancing stretchability and conductivity: Carbon nanotube layer-enhanced non-ionic conductive hydrogels with a sandwich structure. Chem. Eng. J..

[74] Jiang Z., Lv Q., Yan L., Chen X., Zhao F., Pan B., Chen S. (2026). Ionic thermoelectric poly(vinyl alcohol)/gelatin hydrogel for passive multimodal physiological sensing via the sol-gel transition triggered by salting out effect. React. Funct. Polym..

[75] Liang X., Chen S., Liang Y., Wang M., Wang Q., Chen D., Ma X., Zhong H.-J. (2026). Alginate-Based Hydrogels: Recent Progress in Preparation, Property Tuning, and Multifunctional Applications. Gels.

[76] Guan L., Li X., Zhao H. (2026). Conductive Hydrogels: Progress and Prospects in Biomedical Engineering. Macromol. Rapid Commun..

[77] Shen C., Wang Y., Xiao X., Yang R., Chen H., Yuan P., Zhang Y., Lyu G., Shin J., Chen G. (2026). Hydrogels for in vivo biomedical applications: Recent advances and future perspectives. J. Mater. Chem. B.

[78] Zhang K., Huang Y., Han J., Li Z., Wang J., Yang S. (2026). Design and Sensing Applications of Eutectogels: A Review. Materials.

[79] Sun L., Zhang Y., Yang Z., Zhang Y., Dong Q., Zhang Z., Zhang Q. (2026). Recent advances in synthesis and biomedical applications of structurally chiral plasmonic nanomaterials. Coord. Chem. Rev..

[80] Rahman M.S., Helal E., Demarquette N.R. (2026). Recent Advances in Metal-Organic Framework-Integrated Nanocomposite Hydrogels for Sensors and Sensing Systems. SmartMat.

[81] Talipova A.B., Buranych V.V., Savitskaya I.S., Bondar O.V., Turlybekuly A., Pogrebnjak A.D. (2023). Synthesis, Properties, and Applications of Nanocomposite Materials Based on Bacterial Cellulose and MXene. Polymers.

[82] Li Z., Cui C., Yang F., Yuan D., Wang H., Lin Z., Shao L., Wang Q., Jiang W., Shi L. (2026). Loofah-inspired recyclable MXene/polypyrrole fiber-skeleton hydrogels for machine-learning-assisted multimodal flexible sensor. Chem. Eng. J..

[83] Fu H., Li N., Yuna Y., Wang Z., Chain S., Duan T., Xu J. (2026). Metal coordination-hydrogen bonding synergistic cross-linked MXene for 3D-printed micro-supercapacitors in self-powered sensing systems. Chem. Eng. J..

[84] Shi J., Wang S., Wang H., Gu J. (2023). Mechanically Tough and Highly Stretchable Hydrogels Based on Polyurethane for Sensitive Strain Sensor. Polymers.

[85] Akram T., Zhang B., Zhao G., Akram T., Zhang B., Zhao G. (2026). MXene-Polymer Hydrogel Sensors for Next-Generation Advanced Wearable Sensing: From Synthesis to Real World Integration. Adv. Mater. Technol..

[86] Arinova A., Boukhvalov D.W., Umirzakov A., Bondar E., Shongalova A., Mustafa L., Kemelbekova A., Dmitriyeva E. (2025). Synthesis and Studies of PAM-Ag-g/WS2/Ti3C2Tx Hydrogel and Its Possible Applications. Polymers.

[87] Annu, Han S.S., Shin D.K. (2026). Chitosan/MXene hydrogels for biomedical and self-powered health-monitoring bioelectronics: Challenges and strategic solutions. Int. J. Biol. Macromol..

[88] Wei Q., Chu F., Tian C., Ke H., Wang X., Chen X., Qiang L., Xu S. (2026). Robust and anti-swelling MXene composite hydrogel sensors for intelligent gait monitoring via a CNN-LSTM network. RSC Adv..

[89] Li Y., Xuan X., Pan Z., Li Y., Do T.Q., Phan G., Chen H., Thambi T. (2025). A robust and multifunctional conductive double-network hydrogel exhibiting self-healing, anti-freezing, antibacterial, and electromagnetic shielding properties for advanced wearable sensors and biofabrication. Chem. Eng. J..

[90] Tan S.-C., Teng F.-R., Huang Y., Zhang S., Li A.-D. (2026). Ultrastretchable and Self-Healing Polyacrylic Acid Hydrogel Strain Sensor with Pt Nanoparticles-Modified MXene for Machine Learning-Assisted Gesture Recognition. ACS Appl. Nano Mater..

[91] Tan W., Zhalo W. (2020). Designing WS2@Ti3C2Tx heterojunction nanofillers via electrostatic self-assembly for achieving long term corrosion resistance under AHP environment. Mater. Today Nano.

[92] Zhang Y., Wang S., Tian Y., Chen L., Du Y., Su G., Hu Y. (2023). Multi-Physically Cross-Linked Hydrogels for Flexible Sensors with High Strength and Self-Healing Properties. Polymers.

[93] Wang Y., Wang M., Dai Y., Li M., Wang B., Qi K., Ramakrishna S., Ou K. (2026). Carbon Nanotube-Encapsulated Liquid Metal Hydrogel Fibers: An Integrated Conductive, Stretchable, and Adhesive Platform for Multifunctional Sensing. ACS Appl. Mater. Interfaces.

[94] Huang Y., Yan T., Jiang L., Pan W.G., Wang L.W. (2026). Advances in nanofiber hydrogels: Revolutionizing wearable technology applications. Renew. Sustain. Energy Rev..

[95] Anwar M.Z., Kathuria H., Er J.X., Wang Y., Chiu G.N.C. (2026). Hybrid hydrogels for biomedical applications: Addressing challenges in drug delivery through advanced crosslinking and nanocarrier integration. J. Control. Release.

[96] Ampong D.N., Aguekum E., Dzikunu P., Ocloo D., Clottey I., Aggrey P., Nartey M.A., Agyemang F.O., Mensah-Darkwa K., Gupta R.K. (2026). Harnessing hydrogels for electrochemical energy and environmental sustainability. Renew. Sustain. Energy Rev..

[97] Peñalver I., Torres M., Lu L., He C., Xu X. (2026). Dual-Mode Electrical–Optical Nanocomposite Hydrogel with Enhanced Upconversion Luminescence for Strain and pH Sensing. Gels.

[98] Sun X., Barker D., Travas-Sejdic J. (2026). Transient degradable electronics enabled by systems of conducting polymers and natural biopolymers. J. Mater. Chem. C Mater..

[99] Ba Y., Chang Y., Yu Z. (2026). Natural gelatin hydrogel active sensor based on ion–electron dual-pathway synergistic conduction. Flex. Print. Electron..

[100] Li X., Zhou Y., Wei Z., Yin H., Wang P., Li G., Meng C. (2026). High-Performance Hydrogel-Based Temperature Sensor by Integrating Ion Channels and Stabilized Ion Gradients. Adv. Funct. Mater..

[101] Guo Y., Wang Y., Wang H., Hu X., Zhou G., Liu S., Li J. (2026). Highly Flexible, Adhesive, Antimicrobial, and Self-Powered Hydrogel Strain Sensor for Human Motion Monitoring. Biomacromolecules.

[102] Vinnakoti M., Illa R. (2026). Design and Development of PVA/Starch/Sunitinib Malate Composite Hydrogels with Enhanced Mechanical Strength, Conductivity, and Self-Healing Capability for Sensor Applications. J. Appl. Polym. Sci..

[103] Wang X., Cheng J., Han Z., Cheng W., Han G., Wang Y., Wang D. (2026). Superelastic and highly sensitive conductive hydrogel sensor enabled by spatially confined assembly of MXene within bacterial cellulose network. Int. J. Biol. Macromol..

[104] Li S., Wang N., Zhan S., Sheng L., Wang H., Fu Y., Wang B., Liu C., Yang H.Y. (2026). Dynamic hofmeister effect-engineered thermosensitive ionic conductive hydrogel with 3D plasticity and environmental adaptability for wearable strain sensor. J. Colloid Interface Sci..

[105] Wang B., Zhang H., Zhao W., Zhang X., Wang L. (2026). Mechano-nanoconfinement synergy constructs layered rigid-soft interlocked architecture for ultra-strong and resilient hydrogels. Chem. Eng. J..

[106] Nagay B.E., Janghour L.M., El-Khordagui L.K., Akhavan B., Barao V.A.R., Dananjaya V., Abeykoon C., El-Habashy S.E., Dodda J.M. (2026). Multifunctional implantable hydrogels: Smart platforms at the forefront of biomedical innovation. Mater. Today Bio.

[107] Shin M., Lim J., An J., Yoon J., Choi J.W. (2023). Nanomaterial-based biohybrid hydrogel in bioelectronics. Nano Converg..

[108] Duan H., Zhang Y., Zhang Y., Zhu P., Mao Y. (2024). Recent Advances of Stretchable Nanomaterial-Based Hydrogels for Wearable Sensors and Electrophysiological Signals Monitoring. Nanomaterials.

[109] Chauke H., Miya N., Moloto W., Jijana A., Zigyalew Y., Mamo M.D., Machogo-Phao L., Sikhwivhilu L., Ntsendwana B. (2026). Advanced graphene hydrogels in flexible electronics and sensor applications. Graphene Hydrogels: Synthesis, Properties, and Applications.

[110] Asthana N., Khan U.A., Srivastava A., Kumar D., Mishra A.K. (2025). Integration and Characterization of Synthetic Biodegradable Polymer (PVA) with Graphite Oxide (GO) for Performance Assessment in Sustainable Electrochemical Devices. J. Inorg. Organomet. Polym. Mater..

[111] Alotaibi A.M., Alansi A.M., Alade I., Qahtan T.F. (2026). Green nanochemistry approaches for the sustainable synthesis of biomedical nanomaterials and their emerging applications. Adv. Powder Technol..

[112] Lv Y., Li Y., Pan Y., Li Q., Shi C., Gu R., Wei L. (2026). Progress in the application of conductive hydrogels in wound healing: A review. Nanoscale Adv..

[113] Espenti C.S., Mettu M.R., Surendra T.V., Boora S., Kummara M.R., Rao K. (2025). pH-responsive polymer hydrogel nanocomposites for sensor applications: A review. Sens. Actuators A Phys..

[114] Xu H., Zhao J., Feng S., Xu Z. (2026). All-in-one nanocomposite hydrogel bridging wearable sensing and environmental remediation. Microchem. J..

[115] Syed S.M., Kulkarni S., Patil M., Satpute K. (2026). A comprehensive review of green synthesis methods and applications of nanoparticles derived from plant extracts and microorganisms. Discov. Green Chem..

[116] Razzak S.A., Uddin S., Anwarul H., Nawaz A., Siddiquee M.N., Al Bari A., Hossain M.M. (2026). Biomass-derived carbon materials as sustainable platforms for advanced biomedical applications. Environ. Surf. Interfaces.

[117] Chen D., Zhang X., Zhu H., Bai H. (2026). Conductive hydrogels with oriented dual-network structure for flexible strain sensors: Integrating strength, adhesion, and orientation-dependent sensing. Polymer.

[118] Li Z., Shen C., Shin J., Dixit K., Kumar H., Zhang H., Liu D., Lu Q., Chen G., Lee H.J. (2026). Encapsulation of patterned carbon nanotube in PVA-SbQ hydrogels via embedded printing for advanced biocompatible organ patches. Mater. Des..

[119] Hsu C.Y., Alnaimat F., PadmaPria G., Al-Hasnaawei S., Najafloo M., Dehghanipour M., Ray S., Chennakesavulu K., Sharma R., Chauhan A.S. (2026). Smart hydrogel architectures for integrated supercapacitors, sensors, and biomedical technologies. Mater. Today Chem..

[120] Saria M., Hayat S., Ijaz M.U., Saqalein M., Ashraf A., Muzammil S. (2026). Recent advances in graphene hydrogels for biomedical applications. Graphene Hydrogels: Synthesis, Properties, and Applications.

[121] Ahmad A., Nazar M., Reddy A.V.B., Al-Hamouz O.C.S., Hussain S.M.S., Moniruzzaman M. (2026). Prospective routes for the synthesis of graphene-based hydrogels. Graphene Hydrogels: Synthesis, Properties, and Applications.

[122] Kim S., Subba S.H., Kim T.M., Darmawan N.S., Park S.Y. (2026). Light-responsive microporous structure-mediated tunable mechanical and conductive hydrogel for bioelectronic sensor with photothermal therapy. Chem. Eng. J..

[123] Wu Y., Zhao K., Wang J., Li C., Jiang X., Wang Y., Gu X. (2025). Five-Cavity Resonance Inspired, rGO Nano-Sheet Reinforced, Multi-Site Voice Synergetic Detection Hydrogel Sensors with Diverse Self-Adhesion and Robust Wireless Transmissibility. Gels.

[124] Villegas E., Diaz-Barrios A., Gonzalez G., Chimborazo J., Reinoso C., Caetano M., de Lima L., Gardener J., Solorzano G. (2025). Enhanced luminescent properties of carbon quantum dots on thermo-responsive vinylcaprolactam hydrogel matrix. Carbon Trends.

[125] Cui Y., Wang Z., Zhao M., Wang Z., Zong L. (2025). Biomimetic Cellulose Nanocrystals Composite Hydrogels: Recent Progress in Surface Modification and Smart Soft Actuator Applications. Nanomaterials.

[126] Tang Y., Fang Z., Lee H.J. (2024). Exploring Applications and Preparation Techniques for Cellulose Hydrogels: A Comprehensive Review. Gels.

[127] Dhar A.K., Chowdhury N., Biswas O., Mahmood A., Ahmed N., Poudel S., Lucky B.P., Sami S.T., Mazumder J. (2026). Cellulose–graphene composite hydrogels (CGHs). J. Mater. Sci..

[128] Zhang Y., Deng W., Wu M., Rahmaninia M., Xu C., Li B. (2023). Tailoring Functionality of Nanocellulose: Current Status and Critical Challenges. Nanomaterials.

[129] Yang H., Han W., Shi C., Miao D., Wang D., Shi Z., Yang J., Shen J. (2026). Tannic-Ca2+ complex-induced rapid fabrication of anti-freezing and adhesive hydrogels for high-performance strain sensing. Chem. Eng. J..

[130] Gonzalez-Dominguez M., Maruthapandi M., Luong J.H.T. (2026). Bacterial Cellulose Scaffolds for Advanced Wound Care: Immunomodulation, Mixed Biofilms, and Smart Regenerative Dressings. Macromol.

[131] Yu Z., Zeng F., Cai Y., Wang Z., Chen Z., Wang R., Sun Q., Tang X., Shen X. (2025). Dual-responsive carboxymethyl cellulose-based hydrogel sensor with antibacterial activity for wound monitoring and infected wound repair. Chem. Eng. J..

[132] Xu R., Zhang C., Gao Y., Wu X., Quan Y., Zhang Y., Song S., Wei Q. (2025). Mussel-inspired PAM–PDA/Ga^3+^ hydrogels with antibacterial, adhesive and self-healable properties for wearable strain sensors. Polym. Chem..

[133] Nan N., Li Q., Wen X., Jia B., Li H., Bao L. (2026). Carbon-dot nanozyme-empowered responsive hydrogels for smart wound dressing. Chem. Eng. Sci..

[134] Tian B., Ye F., Ma H., Zhao C., Lin Z., Sun Q., Hu X., Ang R. (2026). Thermoelectric composite hydrogels with ionic-electronic transport for bioactive energy harvesting and sensing. Nano Energy.

[135] Wei Y., Cong B., Ji X., Wang D., Hu J., Mou Z., Song H., Zhi Y., Liu Y., Hu J. (2026). Multifunctional carboxymethyl cellulose/polydopamine dual-network conductive hydrogels for motion detection and photothermal therapy. Carbohydr. Polym..

[136] Du X.Y., Song Y.J., Du C., Liu J.D. (2026). Multifunctional microfluidic-directed polymer/hydrogel fabrics towards pH-responsive drug delivery, wound monitoring and wearable sensing. J. Control. Release.

[137] Yao Y., Zhou Y., Lu Y., Zheng H., Zhong W. (2026). Oligopeptide-assisted synthesizing robust, highly compressible, and low-hysteresis multi-level crosslinking zwitterionic polymer hydrogels as wide-temperature range strain sensors. Chem. Eng. J..

[138] Davuluri V., Regala A., Thotakura R. (2026). Strategies for enhancing electrical conductivity in graphene hydrogels. Graphene Hydrogels: Synthesis, Properties, and Applications.

[139] Hublikar L.V., Ganachari S.V. (2026). Functionalization and surface modification of graphene hydrogels. Graphene Hydrogels: Synthesis, Properties, and Applications.

[140] Mampane L., Mamo M.D., Motaung M.P., Jijana A., Sikhwihilu L., Ntsendwana B., Moloto N. (2026). Electrically conducting graphene hydrogels in healthcare applications. Graphene Hydrogels Synthesis, Properties, and Applications.

[141] Du T., Cui T., Yang B., Wu B., Zhang C., Shang K., Yang Y., Wu J., Gao Y., Wang M. (2026). Multifunctional Flexible Sensor Based on P(AA-NIPAM)/PVA Dual-Network Hydrogel for Monitoring Soccer Players’ Motion. Adv. Mater. Technol..

[142] Ren D., Zhang C., Ma T., Wang J., Ju J., Cao X., Yan Y., Li Y., Fan Y., Huang F. (2026). Dechlorination-Triggered Nano-Welding: A Universal Strategy for Ultra-Stretchable, Low-Hysteresis Conductive Elastomer. Adv. Funct. Mater..

[143] Li W., Cai X., He J., Liu H., Gui X., Lin S. (2026). A flexible strain sensor with high adhesion and mechanical enhancement based on PDMS/MWCNTs. Colloids Surf. A Physicochem. Eng. Asp..

[144] Liu M., Chu R., Li G., Song Z., Yu D., Wang H., Liu H., Liu W. (2025). Hydrogel-infiltrated micropatterned nano-carbon aerogel sheet composed of partially carbonized cellulose nanofibers for wearable sensor. Nano Energy.

[145] Wang J., Li W., Liu J., Li J., Wang F. (2025). Supersensitive strain/pressure sensor based on PDA@CNT-K^+^ nanohybrid hydrogel for human motion detection and information transmission. Polymer.

[146] Wang F., Wang J., Li W., Wu Y., Liu J. (2026). High performance hydrogel sensor based on rGO integrated P(AA-HEMA) and starch for motion and temperature monitoring. Carbohydr. Polym..

[147] Sun L.L., Zhao J., Zhao L.J., Sun S.N., Cao X.F. (2026). Sustainable construction of lignin-containing cellulose nanofibrils reinforced antifreeze deep eutectic solvent gel for wearable human motion strain sensors. J. Clean. Prod..

[148] Chen J.Y., Lu B.-C., Zhang B., Chen X.-X., Yao X.-H., Zhang D.-Y., Zhao W.-G. (2026). Hierarchically structured cellulose hydrogel with high stretchability, conductivity, and stability for flexible sensing applications. Int. J. Biol. Macromol..

[149] Liu H., Wang G., Wang M., Lv M., Liu T., Yu M., Lan C. (2026). Synergistic effect of GaInSn liquid metal and MXene in biomimetic sandwich hydrogels: A strategy for high-sensitive, self-healable strain sensors. Chem. Eng. J..

[150] Wang H., Song R., Chen G., Wang F., Wang L., Lei J., Liu J. (2026). Biomass-based dual-network multifunctional hydrogel for durable wearable sensors and emergency hemostasis. Chem. Eng. J..

[151] Chen T., Chen X., Lin Z., Zhang Y., Zhao G., Xu L., Peng X., Wu B. (2026). Biodegradable, Stretchable, and Self-Healing Starch-Based Hydrogel with Intelligent Multi-Bond Network Facilitated by MXene Nanosheets for Multifunctional Wearable Electronics. Adv. Mater..

[152] Qiao Y., Wang Y., Li Y., Liu X., Wang H., Yin X., Zhang S., Wang X., Gu Z., Han P. (2026). Robust PAM/HACC dual-network hydrogel with hydroxylated carbon nanotubes for strain sensing and electromagnetic interference shielding. Colloids Surf. A Physicochem. Eng. Asp..

[153] Chen L., Yu Li X., Su J.L., Xiao Q., Ning Y.M., Yu Z.C., Cui T.J. (2026). Flexible and Robust Metasurface-Based Wearable Sensor for Intelligent Human Monitoring. Adv. Mater..

[154] Peng G., Yuan Y., Tian X., Sun J., Yu H., Cai C. (2026). Stretchable Elastomer Strain Sensor Based on a Three-Dimensional Cross-Linked Conductive Network of Polyaniline/3-Aminobenzenesulfonic Acid-Epoxidized Natural Rubber for Human Motion Monitoring. J. Appl. Polym. Sci..

[155] Zhao W., Geng L., Li J., Zhang H., Shang S., Guo Y., Cheng S., Zhao W. (2025). Flexible piezoresistive sensing device with integrated design and fabrication for health monitoring. Chem. Eng. J..

[156] Li X., Xia Z., Zheng Y., Shang S., Xin B. (2026). MWCNT-reinforced PVA/CS conductive hydrogel for high-sensitivity motion sensing. J. Polym. Res..

[157] Lee J.Y., Tey W.Y., Chai P.V., Quen L.K., Panpranot J., Lee K.M. (2026). Cellulose nanocrystals hydrogel induced by deep eutectic solvent for the fabrication of conductive flexible strain sensors with superior tensile and self-healing properties. Biomass Convers. Biorefin..

[158] Liao L., Ding J., Xiong X., Quan F., Liu X., Zhu X., Chen Z., Li S., Zhu L., Wei B. (2026). Triple-Dynamic-Bond-Engineered Self-Healing Conductive Hydrogels for Deformation-Immune Flexible Supercapacitors and Wearable Epidermal Sensors. Biomacromolecules.

[159] Li S., Ju L., Pan Y., Zhu N., Li J., Wang J., Song Z., Wang L., Shi R., Lin L. (2026). Towards wearable electronic devices: A high linearity bionic flexible stretchable sensor based on MXene/GO nanocomposites and gradient stiffness strategy. Talanta.

[160] Tarashi S., Nazockdast H. (2026). Unveiling the pivotal role of pH in the development of a mechanically strong and adsorptive nanocomposite double-network hydrogel. Mater. Today Commun..

[161] Mu Y., Liu Y., Dong Y., Liu J., Hou M., Sun Y., Li Y., Li J. (2026). Sustainable and multi-stimulus response coal-based carbon quantum dots hydrogel for wearable sensors. Polymer.

[162] Chen C.-T., Guo J.-H. (2026). LCD 3D printing of conductive photo-nanocomposite for a piezoresistive component integrated with wearable rings. Sens. Actuators A Phys..

[163] Liu J., Jia M., Yang J., Wang W., Bai L., Chen H., Wei K., Yang L. (2026). Dual-functional flexible sensor based on CNCs nanocomposite hydrogels for sweat analysis and human motion monitoring. Colloids Surf. A Physicochem. Eng. Asp..

[164] Zhou J., Zheng J., Wang C., Fan M., Xiong F., Li Y., Yang C. (2026). Fabrication of high-toughness PEDOT:PSS-based conductive hydrogel strain/temperature sensors. RSC Adv..

[165] Wang R., Pang H., Guan R., Du W. (2026). Hoffmeister effect assisted the double-network cellulose hydrogel electrolyte with extreme environmental stability and enhanced conductivity for wearable devices and strain sensors. J. Mater. Sci. Technol..

[166] del Río E.P., Esplugues-Lopez A., Heyvaert Y., Jergitsch M., Colombi S., Ahmadi M., Martinez H., Gineba M.-P., Aleman C., Mateos-Timoneda M.A. (2026). Integrating electrical conductivity capability into 3D printed alginate-gelatin hydrogels as skin tissue constructs for temperature sensing. Colloids Surf. B Biointerfaces.

[167] Cao J., Liu J., Wang W., Bai L., Chen H., Wei K., Yang L. (2026). Dynamic network hydrogel with cryogenic mechanical reinforcement, self-healing for motion sensing. Chem. Eng. Sci..

[168] Mao Y., Gao M., Qian C., Zhang N., Miao R., Fan X., Li Y. (2026). Multi-responsive and self-sensing flexible actuators based on conductive polypyrrole/poly(N-isopropylacrylamide) hydrogels. Sens. Actuators A Phys..

[169] Li J., Shi S., Wang W., Bai L., Chen H., Wei K., Yang L. (2025). Seawater-assisting antifreezing hydrogels with functional cellulose nanocrystals for wearable flexible sensors. Chem. Eng. J..

[170] Xu Q., Hou M., Wang L., Zhang X., Liu L. (2023). Anti-bacterial, anti-freezing starch/ionic liquid/PVA ion-conductive hydrogel with high performance for multi-stimulation sensitive responsive sensors. Chem. Eng. J..

[171] Zong S., Wen X., Lei F., Zhu L., Jiang J., Duan J. (2025). Construction of environmentally stable self-adhesive conductive cellulose hydrogel for electronic skin sensor via autocatalytic fast polymerization strategy at room temperature. Int. J. Biol. Macromol..

[172] Liu L., Zhong X., Wang A., Gu Q., Sun C., Wang F., Lei L., Zhang W. (2025). Biodegradable natural hydrogels: Design, crosslinking, and medical applications. Mater. Today Commun..

[173] Jeyalakshmi C., Parameswari M., Vaidehi V., Muthukumaran B., Jayamoorthy K. (2026). Next-generation biodegradable sensors: A roadmap toward zero-waste electronics. Microchem. J..

[174] Kantasiri T., Trisong P., Pimphatam P., Uyama H., Theerakulpisut S., Okhawilai M., Chindaprasirt P., Kasemsiri P. (2026). Green conductive hydrogel from metal–phenolic networks derived from Fe (III) and spent green tea for smart strain sensors and flexible electronics. Mater. Sci. Eng. B.

[175] Ji F., Shang P., Lai Y., Wang J., Zhang G., Lin D., Xu J., Cai D., Qin Z. (2023). Fully Physically Crosslinked Conductive Hydrogel with Ultrastretchability, Transparency, and Self-Healing Properties for Strain Sensors. Materials.

[176] Yang T., Shen T., Duan B., Liu Z., Wang C. (2025). In Vivo Electrochemical Biosensing Technologies for Neurochemicals: Recent Advances in Electrochemical Sensors and Devices. ACS Sens..

[177] Li Y., Pei S., Wang J., Zhang C., Shi B., Luo Z. (2026). Wearable Sensors Fabricated by 3D-Printed Composite Hydrogel with 2D Fillers. Small Methods.

[178] Eckstein S., Benkirane S., Rufo-Martín C., Youssef G. (2026). 3D printing of pseudo-woven continuous carbon fiber meta-skins. Compos. Sci. Technol..

[179] Xu K., Huang B., Zhang X., Ge S.S., Wei X. (2025). 3D Printed Flexible Piezoelectric Sensor with Enhanced Performance for Gait Recognition. ACS Appl. Electron. Mater..

[180] Zhao Q., Liu C., Chang Y., Wu H., Hou Y., Wu S., Guo M. (2023). Low-Temperature 3D Printing Technology of Poly (Vinyl Alcohol) Matrix Conductive Hydrogel Sensors with Diversified Path Structures and Good Electric Sensing Properties. Sensors.

[181] Tang Z.H., Xue S.S., Wang D.Y., Huang P., Li Y.Q., Fu S.Y. (2023). 3D printing of soft and porous composite pressure sensor with monotonic and positive resistance response. Compos. Sci. Technol..

[182] Li M., Wang Y., Wei Q., Zhang J., Chen X., An Y. (2024). A High-Stretching, Rapid-Self-Healing, and Printable Composite Hydrogel Based on Poly(Vinyl Alcohol), Nanocellulose, and Sodium Alginate. Gels.

[183] Zhang P., Teng Z., Yu X., Yang H., Niu J., Wen H., Liu Z., Wang D., Zhang S., Hu Z. (2026). Unibody flexible hydrogel sensor in vivo via NIR three-dimensional printing. Cell Biomater..

[184] Ma H., Su X., Liang J., Liu L., Sun J., Tong J., Lu J., Zhang Y., Lei B., Zhao H. (2025). Bioactive protein/polysaccharide hydrogel functionalized bone implants surface for enhanced osteogenesis. Int. J. Biol. Macromol..

[185] Seneviratne D.M., Whiteside E.J., Windus L.C.E., Burey P., Ward R., Annamalai P.K. (2025). Emerging Biomedical Applications of Sustainable Cellulose Nanocrystal-Incorporated Hydrogels: A Scoping Review. Gels.

[186] Kumar D., Azhar M., Sridhar S.B., Shareef J., Wadhwa T., Malviya R. (2026). Bio-printed cellulose nanocrystal: Processing, fabrication, and biomedical applications. JCIS Open.

[187] Shin M., Lee S., Shin R.S., Choi J.-H., Choi J.-W. (2026). Nanomaterial-Based Muscle Cell/Neural Tissue Biohybrid Robots: From Actuation to Biomedical Applications. Adv. Robot. Res..

[188] Filippi M., Balciunaite A., Georgopoulou A., Paniagua P., Drescher F., Nie M., Takeuchi S., Clemens F., Katzschmann R.K. (2025). Sensor-Embedded Muscle for Closed-Loop Controllable Actuation in Proprioceptive Biohybrid Robots. Adv. Intell. Syst..

[189] Shen C., Wang Y., Yuan P., Wei J., Bao J., Li Z. (2026). Conductive Hydrogels in Biomedical Engineering: Recent Advances and a Comprehensive Review. Gels.

